# Tenodesis yields better functional results than tenotomy in long head of the biceps tendon operations—a systematic review and meta-analysis

**DOI:** 10.1007/s00264-022-05338-9

**Published:** 2022-03-07

**Authors:** Mátyás Vajda, Lajos Szakó, Péter Hegyi, Bálint Erőss, Anikó Görbe, Zsolt Molnár, Kincső Kozma, Gergő Józsa, László Bucsi, Károly Schandl

**Affiliations:** 1 Institute for Translational Medicine, Medical School, University of Pécs, Szigeti út 12, 2nd floor, 7624 Pécs, Hungary; 2 Saint George University Teaching Hospital of County-Fejér, Seregélyesi u. 3., 8000 Székesfehérvár, Hungary; 3 Department of Orthodaedics, Medical School, University of Pécs, Akác u. 1, 7632 Pécs, Hungary; 4 Centre for Translational Medicine, Semmelweis University, Üllői út 26, Budapest, 1085 Hungary; 5 Department of Anaesthesiology and Intensive Therapy and Pain Management, Poznan University of Medical Sciences, 49 Przybyszewskiego St, 60-355 Poznan, Poland; 6 Department of Ophtalmology, Medical School, University of Pécs, Rákóczi út 2, 7623 Pécs, Hungary; 7 Department of Paediatrics, Surgical Division, University of Pécs, József Attila u. 7, 7623 Pécs, Hungary

**Keywords:** Long head of the biceps tendon, LHBT, Tenotomy, Tenodesis, Shoulder arthroscopy, Rotator cuff, Systematic review, Meta-analysis

## Abstract

**Background:**

Pathology of the long head of the biceps tendon (LHBT) is a common disorder affecting muscle function and causing considerable pain for the patient. The literature on the two surgical treatment methods (tenotomy and tenodesis) is controversial; therefore, our aim was to compare the results of these interventions.

**Methods:**

We performed a meta-analysis using the following strategy: (P) patients with LHBT pathology, (I) tenodesis, (C) tenotomy, (O) elbow flexion and forearm supination strength, pain assessed on the ten-point Visual Analog Scale (VAS), bicipital cramping pain, Constant, ASES, and SST score, Popeye deformity, and operative time. We included only randomized clinical trials. We searched five databases. During statistical analysis, odds ratios (OR) and weighted mean differences (WMD) were calculated for dichotomous and continuous outcomes, respectively, using the Bayesian method with random effect model.

**Results:**

We included 11 studies in the systematic review, nine of these were eligible for the meta-analysis, containing data about 572 patients (279 in the tenodesis, 293 in the tenotomy group). Our analysis concluded that tenodesis is more beneficial considering 12-month elbow flexion strength (WMD: 3.67 kg; *p* = 0.006), 12-month forearm supination strength (WMD: 0.36 kg; *p* = 0.012), and 24-month Popeye deformity (OR: 0.19; *p* < 0.001), whereas tenotomy was associated with decreased 3-month pain scores on VAS (WMD: 0.99; *p* < 0.001). We did not find significant difference among the other outcomes.

**Conclusion:**

Tenodesis yields better results in terms of biceps function and is non-inferior regarding long-term pain, while tenotomy is associated with earlier pain relief.

**Supplementary Information:**

The online version contains supplementary material available at 10.1007/s00264-022-05338-9.

## Introduction

The biceps brachii muscle has a proven function in forearm supination and elbow flexion [1]. The separate role of the long head of the biceps tendon (LHBT) is still debated. Cadaver studies [2–6] suggest that the LHBT plays an essential role in the stability of the glenohumeral joint, while the results of in vivo studies are controversial [7–9].

The pathology of the LHBT includes inflammation, partial or complete rupture (including SLAP lesions (superior labrum anterior and posterior)), and instability [1], which can lead to anterior shoulder pain or diminished function [10]. These lesions are often associated with other shoulder pathology, such as rotator cuff (RC) tears [11–15].

In patients undergoing RC repair, the incidence of LHBT pathology shows great heterogeneity throughout the different studies: 36.1–82% [13, 14].

Besides conservative therapy, surgery plays an important role in the treatment. The most used methods are tenotomy and tenodesis; however, there is more than one surgical approach in both groups. Tenotomy is the more straightforward method, where the tendon is released from the supraglenoid tubercle [16]. This can be performed with or without creating a funnel-shaped proximal stump [17] or releasing the LHBT with a portion of the superior labrum [18]. Tenodesis can be performed arthroscopically or through an open approach, and the tendon may be fixed to multiple anatomical locations, such as soft tissue or bone. The site can also be suprapectoral or subpectoral [19]; the fixation may involve suturing to tendons, interference screw, bone tunnels, keyholes, suture anchors, and suture buttons [10, 20, 21].

Some studies have results supporting the beneficial nature of tenodesis [22–27], while others suggest that there is no relevant difference in functional outcomes when comparing tenotomy to tenodesis [17, 28–33].

The previous meta-analyses either did not reach a firm conclusion [34] or included cohort studies [35–41].

Due to the controversial results of clinical trials and limitations of previous meta-analyses, we aimed to provide the most comprehensive analysis to date comparing tenodesis to tenotomy in managing LHBT pathologies.

## Methods

We used the Preferred Reporting Items for Systematic Reviews and Meta-Analysis (PRISMA) statement [42] to report our research.

### Protocol

We registered our research protocol on PROSPERO in advance under the registration number CRD42021244613. There were no protocol deviations.

### Search strategy, inclusion, and exclusion criteria

While stating our clinical question, we used the PICOTS framework. P (population) were the patients who have undergone LHBT operations, I (intervention) was tenotomy, our C (comparison) was tenodesis, and our outcomes were the following: pain on the ten-point Visual Analog Scale (VAS), bicipital cramping pain events, bicipital groove pain events, Constant score (range: 0–100), American Shoulder and Elbow Surgeons (ASES) score (range: 0–100), Simple Shoulder Test (SST) score (range: 0–12), operative time in minutes, elbow flexion strength, forearm supination strength, and Popeye deformity events. Regarding T (timing), we statistically analysed every outcome when at least three studies reported them at the same time point. If an outcome did not qualify for quantitative synthesis, we included it only in the systematic review section. The S (study type) was randomized controlled trials (RCTs).

On 28 November 2020, we conducted a systematic search using the databases of MEDLINE (via PubMed), Embase, Cochrane Central Register of Controlled Trials (CENTRAL), Web of Science, and Scopus, using the following search key: “bicep* AND teno*”. We used the “all fields” option (or the equivalent of it) in the first four databases, while in Scopus we used the “Article title, Abstract, Keywords” search field. We applied no filters in any of the databases.

Our inclusion criteria were the following: RCTs, comparing tenotomy and tenodesis and reporting on the outcomes of interest.

Our exclusion criteria were the following: review, meta-analysis, cohort study, case report, surgical technique description, studies comparing different submodalities (for example, different tenodesis techniques), distal biceps tear, biomechanical study, cadaver study, and animal study.

### Selection and data extraction

We used EndNote X9 (Clarivate Analytics, Philadelphia, PA, USA) for the selection process. After removing the duplicates, two independent review authors (M.V., S.L.) performed the selection, first by title, then abstract, and finally by full text. Following every step of the selection, Cohen’s kappa was calculated to assess the agreement between the two investigators with the following parameters: 0.00–0.20 no agreement, 0.21–0.39 minimal agreement, 0.40–0.59 weak agreement, 0.60–0.79 moderate agreement, 0.80–0.90 strong agreement, and above 0.90 almost perfect agreement [43]. We screened the references of the eligible records for possible additional articles to include in the meta-analysis. The same two review authors conducted data extraction using a pre-specified Excel sheet (Office 2016, Microsoft, Redmond, WA, USA). We gathered data from the articles about the first author, year of publication, country, study design, demographic data, indication of the surgery, surgical methods, and outcomes that we presented. If the strength measurement results were reported in Newton (N), we converted them to kilogram (kg) using an online calculator (calculator-converter.com). If the studies did not report the Strength Index (SI) but did report the strength measurement result of both sides, we calculated SI from them.

Two independent review authors (M.V., L.S.) resolved the disagreements by consensus regarding both the selection and the data extraction process.

### Statistical analysis

For dichotomous outcomes, odds ratios (ORs) with their 95% confidence intervals (CI) were calculated from the original raw data of the articles. We decided to use continuity correction [44] in case of the number of reported bicipital cramping pain events, final data outcome as we observed zero events in some studies. For continuous outcomes, weighted mean differences (WMDs) with 95% CI were calculated from the original raw data of the articles except in some cases where standard deviations (SDs) and means were calculated from the minimum, median, maximum, and sample size according to Wan’s method [45]. The random effect model by DerSimonian and Laird [46] was applied in all cases, with the estimate of heterogeneity. Following the Cochrane Handbook, the *I*^2^ values were considered moderate heterogeneity between 30 and 50%, substantial heterogeneity between 50 and 75%, and considerable heterogeneity higher than 75%. We used forest plots to display the results graphically. When it was statistically possible, we performed a trial sequential analysis (TSA) [47] to confirm the statistical reliability of the data with the calculation of the required information size by adjusting the significance level for sparse data.

We statistically analysed and compared every outcome when at least three studies reported them at the same time point. To provide a clear picture of the available data, we present the individual results of all included studies, comparing the two surgical methods in the systematic review section.

All data management and statistical analysis were performed with Stata (version 16.0, StataCorp) and TSA (trial sequential analysis tool from Copenhagen Trial Unit, Centre for Clinical Intervention Research, Denmark).

### Risk of bias assessment and quality of evidence

We performed the risk of bias assessment for every examined outcome according to the Cochrane recommendation using the RoB 2: A revised Cochrane risk of bias tool for randomized trials [48].

To assess the certainty of the evidence, we used the Grading of Recommendations Assessment, Development, and Evaluation (GRADE) system [49] and classified our results into four levels: high, moderate, low, and very low certainty of evidence.

Two independent review authors (M.V. and L.S.) performed the risk of bias and certainty of evidence assessments. The disagreements were resolved by consensus.

## Results

### Search and selection

**Fig. 1:**
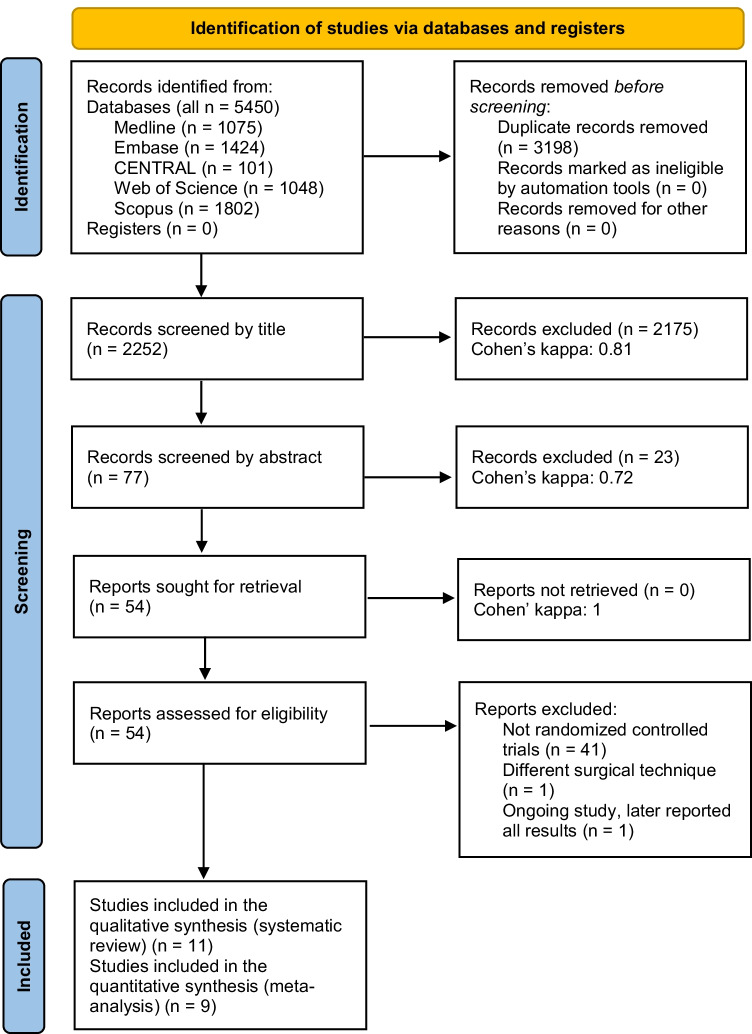
A Preferred Reporting Items for Systematic Reviews and Meta-Analysis (PRISMA) flow chart representing the search and selection process

The summary of our selection process, including the Cohen’s kappa for each step, is shown in Fig. 1. We identified 5450 records in the five databases. After completing the selection process, we were left with nine eligible full-text articles in the meta-analysis [50–58] and eleven studies in the systematic review section [50–60].

### Characteristics of the studies included

We summarized the basic characteristics of the included studies (shown in Table 1). All the included studies were RCTs, and ten of them compared tenotomy to tenodesis [50–55, 57–60]. We included nine studies and 572 participants in the meta-analysis, 293 in the tenotomy group and 279 in the tenodesis group. Two studies ([59, 60]) did not have outcomes with a comparable matching time point; therefore, we were only able to include these in the systematic review section.

**Table 1:** Characteristics of the included studies

First author, year	Study design	Country	Age (mean)	Sex (female % of total)	Number of patients	Follow-up time in months	Inclusion criteria	Type of TT	Type of TD
Belay et al. (2019) [50]	Randomized, controlled, patient-blinded, single-centre	UK	TT: 57.7 TD: 52.9	TT: 5 TD: 14.3	TT: 20 TD: 14	TT: 24 TD: 24	LHBT pathology confirmed with imaging and physical examination (RCRs not excluded, but also not necessary)	ASC scissors: LHBT cut from superior labrum	ASC, interference screws
Castricini et al. (2018) [51]	Randomized, controlled, assessor-blinded, single-centre	Italy	TT: 59.9 TD: 57.1	TT: 54.8 TD: 70.8	TT: 31 TD: 24	TT: 24 TD: 24	Grade I or II full-thickness reparable supraspinatus tendon tear with a LHBT lesion, patients over 40 years old	ASC, releasing of the LHBT from its insertion on the superior glenoid labrum with electrocautery	ASC, interference screws
De Carli et al. (2012) [59]	Randomized, controlled, single-centre	Italy	TT: 59.6 TD: 56.3	TT and TD reported together: 26	TT: 30 TD: 35	TT: 23 * TD: 25 *	Small to large rotator cuff tear and the presence of an associated degenerative lesion of the LHBT, patients younger than 65	ASC, scissors were used to sever the tendon at its junction with the superior labrum	ASC, suturing the LHB to cuff tendons
García-Rellan et al. (2020) [52]	Randomized, controlled, multi-centre	Spain	TT: 54.7 TD: 50.73	TT: 0 TD: 0	TT: 23 TD: 18	TT: 12 TD: 12	Diagnosis of LHBT pathology in men between 40 and 65 years of age, (RCRs not excluded, but also not necessary)	ASC, sectioning the LHBT near of its insertion with an electrocoagulator	ASC, interference screws
Hufeland et al. (2019) [53]	Randomized, controlled, examiner-blinded, single-centre	Germany	TT: 52.8 TD: 51.5	TT: 63.64 TD: 22.22	TT: 11 TD: 9	TT: 12 TD: 12	Isolated SLAP lesion type II–IV, 40–70 years of age (full thickness rotator cuff tear excluded)	ASC, transecting the tendon directly at the SLAP complex with an angulated punch	ASC, interference screws
Lee et al. (2016) [60]	Randomized, controlled, double-blinded, single-centre	Republic of Korea	TT: 62.8 TD: 62.9	TT: 80.357 TD: 75	TT: 56 TD: 72	TT: 25.1# TD: 19.7#	Symptomatic LHBT partial tear and small—to medium-sized rotator cuff tears that required surgical repair, after at least one month of unsuccessful conservative therapy	ASC, funnel-shaped tenotomy: dividing the LHBT at its proximal origin of the labrum	ASC, interference screws
MacDonald et al. (2020) [54]	Randomized, controlled, double-blinded, multi-centre	Canada	TT: 56.3 TD: 58.7	TT: 21.05 TD: 17.54	TT: 57 TD: 54	TT: 24 TD: 24	Patients over 18 years old with intraoperative confirmation of a lesion of the LHBT (RCRs not excluded, but also not necessary)	ASC, LHBT was detached from its proximal anchor to the superior labrum	ASC, interference screws (n = 37), open subpectoral approach with a button (n = 17)
Mardani et al. (2018) [55]	Randomized, controlled, single-centre	Iran	TT: 54.5 TD: 55.5	TT: 31 TD: 33.3	TT: 29 TD: 33	TT: 24 TD: 24	Patients aged 45 to 60 years, arthroscopic RCR with positive biceps test before surgery, and intraoperatively confirmed LHBT pathology	ASC, with the use of a forceps	ASC, reabsorbable interference screw
Oh et al. (2016) [56]	Randomized, controlled, examiner-blinded, single-centre	Republic of Korea	TT: 61.04 TD: 56.61	TT: 66.67 TD: 32.26	TT: 27 TD: 31	TT: 21.98 TD: 21.46	Rotator cuff tear in addition to an intraoperatively confirmed SLBC lesion (type II SLAP lesion, partial tear of LHBT, partial biceps pulley tear)	ASC, scissors at the junction between the biceps tendon and superior labrum	ASC, suture anchor
Zhang et al. (2015) [57]	Randomized, controlled, examiner-blinded, single-centre	China	TT: 61* TD: 61*	TT: 54.25 TD: 52.7	TT: 77 TD: 74	TT: 25* TD: 25*	Patients affected by both rotator cuff tears and LHBT pathologies, age: older than 55	ASC, the tendon was debrided and cut as close as possible to the labrum	ASC, suture anchor
Zhang et al. (2019) [58]	Randomized, controlled, single-centre	China	TT: 62.2 TD: 60.5	TT: 66.67 TD:63.64	TT: 18 TD: 22	TT and TD reported together: 14.3#	Confirmed LHBT pathology, at least six months of unsuccessful conservative therapy, age: between 50 and80 years (RCRs were excluded)	ASC, cut the LHBT at the superior labrum	ASC, suture anchor

^*^Median

^#^Mean

*TT* tenotomy, *TD* tenodesis, *LHBT* long head of the biceps tendon, *RCR* rotator cuff repair, *ASC* arthroscopy, *LHBT* pathology included: degenerative tear, partial rupture, subluxation, dislocation, tenosynovitis, hypertrophy, superior labral tear from anterior to posterior (SLAP) lesions, partial biceps pulley tear

All studies included patients with LHBT pathology; nine of the eleven studies [50–52, 54–57, 59, 60] also included patients with concomitant rotator cuff tear, while two [53, 58] excluded them.

Tenotomy was performed arthroscopically in all studies. Tenodesis was also performed arthroscopically, except in the case of 31.5% of patients (17 out of 54) in the study of MacDonald et al. [54], where surgeons used an open subpectoral approach.

The follow-up times were different in the studies, mostly between 12 and 24 months, with some variation. The evaluation times of several outcomes were also different.

### Meta-analysis results

#### Post-operative function

**Fig. 2:**
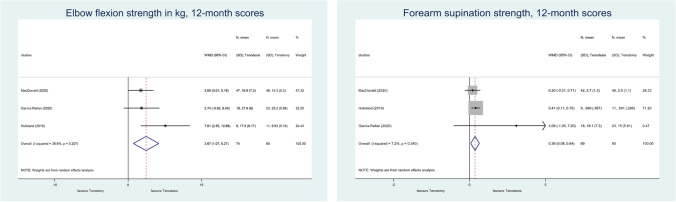
A forest plot that compares the results of elbow flexion strength measurements in kg in tenotomy and tenodesis at the 12-month follow-up and the results of the 12-month forearm supination strength levels of tenotomy and tenodesis. The black diamonds represent the effect of individual studies, and the vertical lines show the corresponding 95% confidence intervals (CI). The size of the grey squares reflects the weight of a particular study. The blue diamond reflects the overall or summary effect. The outer edges of the diamonds represent the CIs

The analysis of elbow flexion strength in kg at the 6-month follow-up showed no statistically significant difference [51, 53, 54] (WMD, 2.82; 95% CI, − 1.79–7.22; *p* = 0.237; *I*^2^ = 71.7%; low grade of evidence) (Supplementary Fig. 1). When comparing the results, the 12-month elbow flexion scores in kg showed statistically significant difference in favour of tenodesis [52–54] (WMD, 3.67; 95% CI, 1.07–6.27; *p* = 0.06; *I*^2^ = 36.6%; moderate grade of evidence) (Fig. 2). Analysis of the 12-month forearm supination strengths also resulted in statistically significant difference [52–54] (WMD, 0.36; 95% CI, 0.08–0.64; *p* = 0.012; *I*^2^ = 7.2%; low grade of evidence) (Fig. 2).

We were able to analyse the Constant score in three studies at the six month follow-up [51, 53, 55] (WMD, 0.78; 95% CI, − 2.44–4.00; *p* = 0.634; *I*^2^ = 27.7%; moderate grade of evidence) (Supplementary Fig. 2) and three studies at the 12-month follow-up time [52, 53, 55] (WMD, 2.26; 95% CI, − 1.12–5.65; *p* = 0.190; *I*^2^ = 59.1%; low grade of evidence) (Supplementary Fig. 3). Neither result showed a statistically significant difference between the two groups. The study of Lee et al. [60] also reported the six month and 12-month Constant scores, but it was not possible to analyse these outcomes due to a lack of data.

#### Post-operative pain

**Fig. 3:**
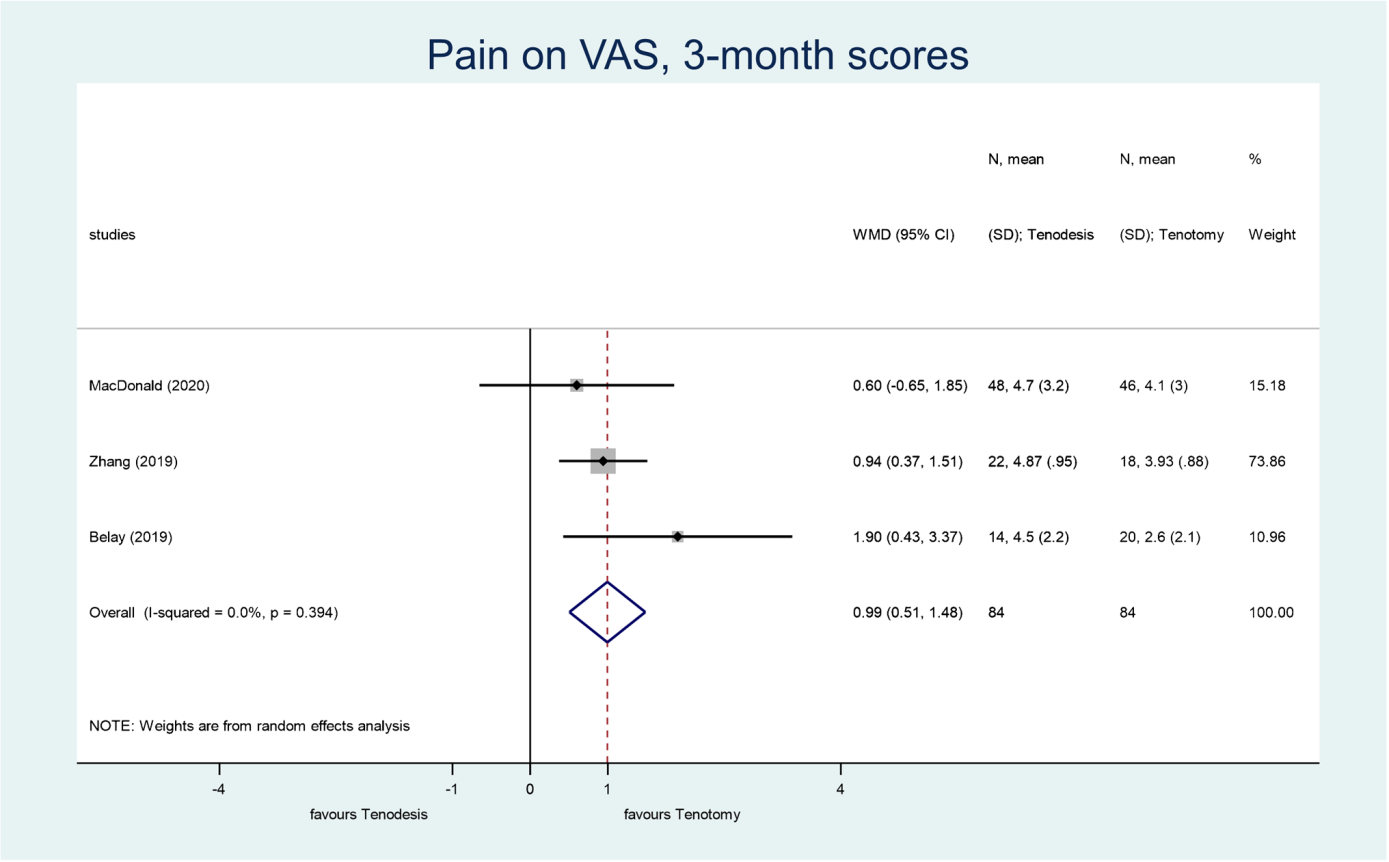
A forest plot that compares the level of postoperative pain on the Visual Analog Scale (VAS) in tenotomy and tenodesis, measured three months post-operatively. The black diamonds represent the effect of individual studies, and the vertical lines show the corresponding 95% confidence intervals (CI). The size of the grey squares reflects the weight of a particular study. The blue diamond reflects the overall or summary effect. The outer edges of the diamonds represent the CIs

Three studies reported three month pain scores on the ten-point VAS [50, 54, 58] (WMD, 0.99; 95% CI, 0.51–1.48; *p* < 0.001; *I*^2^ = 0.0%; high grade of evidence) (Fig. 3). The difference was significant in favour of tenotomy, therefore, leading to the conclusion that there is earlier pain relief with tenotomy than with tenodesis. Four studies reported the 6-month [51, 54, 55, 58] (WMD, 0.05; 95% CI, − 0.21–0.30; *p* = 0.724; *I*^2^ = 0.0%; moderate grade of evidence) (Supplementary Fig. 4), 12-month [52, 54, 55, 58] (WMD, 0.19; 95% CI, − 0.26–0.63; *p* = 0.411; *I*^2^ = 80.1%; very low grade of evidence) (Supplementary Fig. 5), and 24-month [50, 51, 54, 55] (WMD, 0.01; 95% CI, − 0.04–0.07; *p* = 0.637; *I*^2^ = 0.0%; moderate grade of evidence) (Supplementary Fig. 6) pain scores on VAS (different studies reported it at different time points), and we found no significant difference at these time points. The study of Lee et al. [60] also reported the three month, six month, and 12-month level of pain, but it was not possible to analyse these outcomes due to lack of data.

The analysis of bicipital cramping pain events showed no significant difference at 6 months [51, 53, 56] (OR, 0.92; 95% CI, 0.09–9.07; *p* = 0.943; *I*^2^ = 47.8%; moderate grade of evidence) (Supplementary Fig. 7).

#### Popeye deformity

**Fig. 4:**
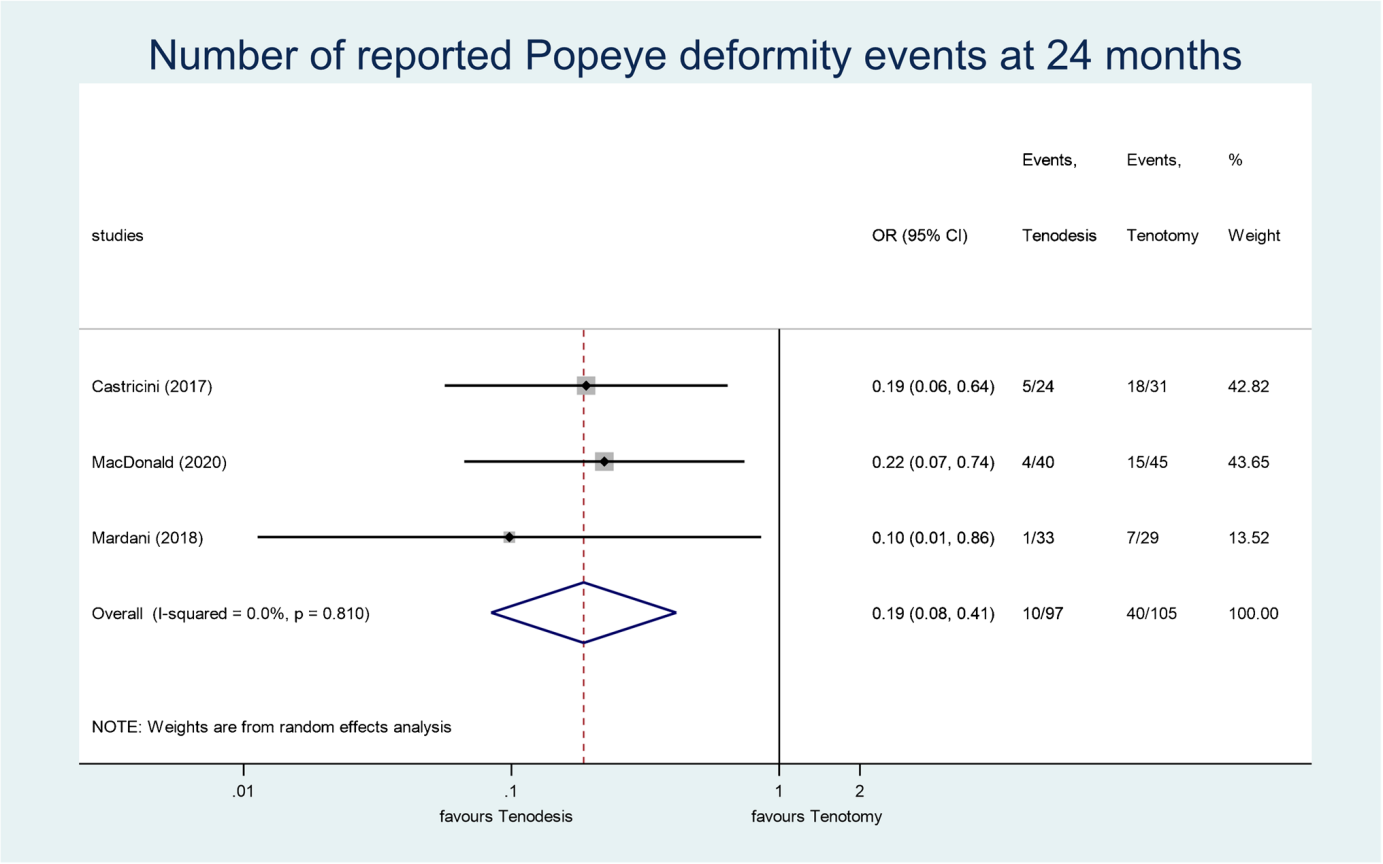
A forest plot that compares the occurrence of Popeye deformity in tenotomy and tenodesis, measured 24 months post-operatively. The black diamonds represent the effect of individual studies, and the vertical lines show the corresponding 95% confidence intervals (CI). The size of the grey squares reflects the weight of a particular study. The blue diamond reflects the overall or summary effect. The outer edges of the diamonds represent the CIs

Three studies [51, 54, 55] reported the occurrence of Popeye deformity at the 24-month check-up. The difference between tenotomy and tenodesis was significant in this outcome in favour of tenodesis (OR, 0.19; 95% CI, 0.08–0.41; *p* < 0.001; *I*^2^ = 0.0%; moderate grade of evidence) (Fig. 4).

#### Operative time

When comparing the operative time (measured in minutes) of tenotomy and tenodesis, we found no statistically significant difference [54, 57, 58] (WMD, 17.15; 95% CI, − 2.05–36.35; *p* = 0.080; *I*^2^ = 97.5%; very low grade of evidence) (Supplementary Fig. 8).

#### TSA (trial sequential analysis)

The results of our TSA are depicted in Supplementary Figs. 9–16. Due to lack of data, TSA was not possible for the following outcomes: 6-six month Constant scores, six month VAS pain scores, 24-month VAS pain scores, and bicipital cramping pain events at six months post-operatively.

#### Systematic review results

Eight studies reported the elbow flexion strength levels [51–54, 56, 57, 59, 60], six studies reported the forearm supination strength levels [52–54, 56, 57, 60], seven studies reported the Constant score [51–53, 55, 57, 59, 60], five papers included the ASES score [50, 53, 54, 56, 60], and three studies reported the SST scores [53, 55, 59]. Nine studies reported pain levels [50–52, 54–58, 60], six studies reported the number of bicipital cramping pain events [51–53, 55–57], and three studies reported the number of bicipital groove pain events [50, 52, 56]. All studies reported the Popeye deformity outcome [50–60]. The article of Lee et al. [60] reported the 3-month, 6-month, 12-month, and final data of the Constant score, ASES score, and level of pain, but it was not possible to analyse these outcomes due to lack of data.

The summary of calculated odds ratios and weighted mean differences for the outcomes that were not eligible for the meta-analysis are shown in Table 2.

**Table 2:** Systematic review: comparing the final data in the individual articles

	Elbow flexion strength in kg (TT/TD)	Elbow flexion strength in SI (TT/TD)	Forearm supination strength in SI (TT/TD)	Constant score (TT/TD)	ASES score (TT/TD)	SST score (TT/TD)	Pain on VAS (TD/TT)	Number of reported bicipital cramping pain events (TD/TT)	Number of reported bicipital groove pain events (TD/TT)	Number of reported Popeye deformity events (TD/TT)
Measure of effect	WMD (95% CI)	WMD (95% CI)	WMD (95% CI)	WMD (95% CI)	WMD (95% CI)	WMD (95% CI)	WMD (95% CI)	OR (95% CI)	OR (95% CI)	OR (95% CI)
Belay (2019) [50]	n.a	n.a	n.a	n.a	− 8.22 (− 22.09, 5.65)	n.a	− 0.82 (− 2.25, 0.61)	n.a	1.21 (0.27, 5.40)	0.23 (0.02, 2.24)
Castricini (2017) [51]	− 3.50 (− 7.52, 0.52)	n.a	n.a	− 0.8 (− 4.66, 3.06)	n.a	n.a	0.00 (− 1.04, 1.04)	1.00 (0.02, 53.90)	n.a	0.19 (0.06, 0.64)
De Carli (2012) [59]	− 0.10 (− 1.48, 1.28)	0.03 (− 0.73, 0.79)	n.a	2.6 (0.21, 4.99)	n.a	1.1 (0.47, 1.73)	n.a	n.a	n.a	0.07 (0.00, 0.52)
García-Rellan (2020) [52]	2.74 (− 0.92, 6.40)	n.a	n.a	0.80 (− 1.29, 2.89)	n.a	n.a	1.02 (0.41, 1.63)	1.71 (0.33, 8.94)	1.73 (0.39, 7.72)	0.10 (0.02, 0.52)
Hufeland (2019) [53]	7.61 (2.55, 12.66)	0.09 (− 0.74, 0.92)	0.13 (− 0.69, 0.95)	10.07 (2.18, 19.22)	18.3 (4.38, 32.22)	1.20 (− 0.29, 2.69)	n.a	1.00 (0.02, 56.40)	n.a	0.33 (0.03, 3.93)
Lee (2016) [60]	n.a	− 0.01 (− 0.03, 0.01)	0.18 (0.15, 0.21)	n.a	n.a	n.a	n.a	n.a	n.a	0.24 (0.07, 0.80)
MacDonald (2020) [54]	− 1.00 (− 3.76, 1.76)	n.a	n.a	n.a	− 2.90 (− 10.57, 4.77)	n.a	− 0.60 (− 1.76, 0.56)	n.a	n.a	0.22 (0.07, 0.74)
Mardani (2018) [55]	n.a	n.a	n.a	1.84 (− 0.41, 4.09)	n.a	0.28 (− 0.12, 0.68)	0.01 (− 0.04, 0.07)	0.03 (0.00, 0.57)	n.a	0.10 (0.01, 0.86)
Oh (2016) [56]	n.a	0.06 (− 0.16, 0.29)	0.24 (0.01, 0.47)	n.a	1.44 (− 2.85, 5.73)	n.a	− 0.09 (− 0.57, 0.39)	0.87 (0.05, 14.56)	1.13 (0.37, 3.46)	0.59 (0.19, 1.81)
Zhang (2015) [57]	n.a	0.00 (− 0.06, 0–06)	0.00 (− 0.05, 0.05)	0.90 (0.01, 1.79)	n.a	n.a	0.10 (− 0.34, 0.54)	1.00 (0.02, 51.09)	n.a	0.28 (0.06, 1.38)
Zhang (2019) [58]	n.a	n.a	n.a	n.a	n.a	n.a	− 0.13 (− 0.44, 0.18)	n.a	n.a	0.03 (0.00, 0.52)

*TT* tenotomy, *TD* tenodesis, *ASES* American Shoulder and Elbow Surgeons, *SST* Simple Shoulder Test, *VAS* Visual Analog Scale, *SI* Strength Index, *kg* kilogram, *OR* odds ratio, *WMD* weighted mean difference, *n.a*. not available

#### Risk of bias assessment and quality of evidence

A summary of the risk of bias assessment is shown in Supplementary Figs. 17–38. The Popeye deformity was the only outcome that all studies reported. In this analysis we found four studies with high risk of bias [55, 58–60], six studies carried “some concerns” [50–53, 56, 57], while one study resulted in low risk of bias [54]. Lower grades were mostly due to the unclear randomization process, the lack of blinding, and the missing trial protocols.

The results of the GRADE analysis are shown for every outcome in the results section. A detailed description of the quality of evidence is found in Supplementary Table 1.

## Discussion

The earlier meta-analyses also included non-randomized trials [35–41] with the exception of Ahmed et al. [34]; hence, their results must be regarded with caution.

Biceps brachii has an essential role in elbow flexion strength. For this reason, we decided to choose this as one of the primary outcome parameters. Even though our analysis did not significantly differ at the 6-month follow-up, at 12 months, the elbow flexion strength was significantly better in the tenodesis group. To our knowledge, this result is a novelty compared to the results of previous meta-analyses that examined this particular outcome [34, 37–40]. Nevertheless, our TSA indicates that further RCTs are needed in the case of the six month results. Even though the required sample size was reached for the 12-month results, potential spurious significance was present; thus, this should be considered inconclusive according to the TSA result. If we consider the results of the individual studies included in the systematic review, we are left with mixed results, but due to the differences in time points, we could not perform more statistical comparisons.

Another major role of the biceps brachii is forearm supination. Our results showed a statistically significant difference between the 12-month supination strength results in favour of tenodesis, contradicting the literature so far [34, 37–40]. According to our trial sequential analysis, further clinical trials are needed to reach a more certain result. Examining the final data from the individual studies, we discovered a tendency in favour of tenodesis.

The Constant score is a widely accepted scoring system used to evaluate post-operative function after shoulder operations. However, it is not specific to biceps function but was designed to assess the overall functional state of the shoulder [62]. Although we found no significant difference between the Constant scores (6 months, 12 months post-operatively), if we add the systematic review results, there is a trend suggesting that tenodesis might lead to better post-operative scores than tenotomy. This result is in accordance with the previous meta-analyses, where they either found statistically significant difference without reaching the minimal clinically important difference [63] (MCID) [34–36, 38–41] or did not find any significant differences when comparing the two methods [37].

From the patient’s perspective, post-operative pain might be the strongest quality measure after surgery. We could analyse the degree of pain as the VAS indicated at three, six, 12, and 24 months after surgery. The difference was significant only at the three month follow-up in favour of tenotomy. The TSA for this outcome showed that no further studies are needed to confirm the result. Thus we can conclude that patients experience less pain three months after tenotomy than those who underwent tenodesis. Despite this, we found no significant differences between the two methods in the long term. Out of the meta-analyses that examined pain on VAS [34, 38–40], only Ahmed et al. [34] evaluated more time points (6, 12, 24 months), but they did not find significant differences between tenotomy and tenodesis. The systematic review results did not suggest any strong tendency toward the preference of tenotomy or tenodesis.

According to some previous articles, one of the drawbacks of tenotomy is that it leads to a higher incidence of cramping pain events [35, 37]. The results of our analysis at the six month follow-up do not support this assumption and are in accord with those analyses which found no difference between tenotomy and tenodesis [34, 36, 38–41]. The results remained the same after we evaluated the data of the systematic review.

In a recent study on 1723 patients, tenotomy was associated with a higher incidence of Popeye deformity than tenodesis [23]. Our results confirmed this data: we also found a significant difference between the two groups in favour of tenodesis, in accordance with earlier meta-analyses [34–41]. The TSA showed that no further clinical trials are needed to confirm this result.

Surgical times can vary greatly for various reasons, including concomitant procedures such as rotator cuff repair and the surgical team’s experience. According to a recent systematic review and meta-analysis, shorter operative time is one of the advantages of tenotomy [35]. Surprisingly, even though all of the included RCTs that examined this outcome [54, 57, 58] found that tenodesis requires more time to perform, the result of our analysis showed no statistically significant difference between tenotomy and tenodesis in this regard. Considering the results established in the literature and the conflicting result of our TSA, no conclusion can be drawn on this topic at present.

### Strengths and limitations

This meta-analysis from nine studies has considerable strengths. Unlike previous analyses, a strict methodology was applied with outcomes assessed only at the same time points. Since we only included randomized controlled trials, this analysis portrays the highest level of achievable evidence on this topic. Trial sequential analyses were performed to assess whether further clinical trials are needed. It was deemed conclusive regarding three month pain levels on the VAS and Popeye deformity at the 24-month follow-up outcomes.

Our meta-analysis had some limitations, including the small sample size that influenced some of the TSA results. In addition, the indication for treatment differed among the included trials, and there was heterogeneity among the studies regarding intervention submodalities and rehabilitation protocols. In some cases, standard deviations (SDs) and means were calculated from the minimum, median, maximum, and sample size. TSA was not conclusive in the following outcomes: six month elbow flexion strength in kg, 12-month elbow flexion strength in kg, 12-month forearm supination strength in kg, 12-month Constant score, 12-month pain levels on the Visual Analog Scale, and operative time in minutes.

We suggest conducting further randomized controlled trials focusing on elbow flexion strength, forearm supination strength, pain, and operative time, as these were deemed inconclusive based on our TSA. When designing an RCT, exact time points regarding the assessment of outcomes are required. The importance of biceps function-specific outcomes such as flexion and supination strength should be highlighted and should be focused on by further RCTs. The use of LHB score [61] might be beneficial in studies focusing on LHBT treatment methods, since it is specific to biceps, unlike the score systems most studies use (Constant, ASES, SST, UCLA (University of California at Los Angeles), etc.). Creating and reporting subgroups would be beneficial (i.e., a group with concomitant rotator cuff surgery and a group without it or comparing different tenotomy methods with the potential for autotenodesis).

## Conclusions

Based on our results, tenodesis should be preferred over tenotomy due to a less frequent occurrence of Popeye deformity, better postoperative biceps function, and the non-inferior nature of tenodesis regarding long-term pain.

## Supplementary Information

Below is the link to the electronic supplementary material.

Supplementary Table 1 Summary of the certainty of evidence according to the GRADE (DOCX 21 KB)

Supplementary Fig. 1 A Forest plot that compares the results of elbow flexion strengthmeasurements in kilogram (kg) in tenotomy and tenodesis 6 months postoperatively. The blackdiamonds represent the effect of individual studies, and the vertical lines show the corresponding95% confidence intervals (CI). The size of the grey squares reflects the weight of a particularstudy. The blue diamond reflects the overall or summary effect. The outer edges of the diamondsrepresent the CIs (PNG 100 KB)

Supplementary Fig. 2 A Forest plot that compares the 6-month Constant scores of tenotomyand tenodesis. The black diamonds represent the effect of individual studies, and the vertical linesshow the corresponding 95% confidence intervals (CI). The size of the grey squares reflects theweight of a particular study. The blue diamond reflects the overall or summary effect. The outeredges of the diamonds represent the CIs (PNG 94 KB)

Supplementary Fig. 3 A Forest plot that compares the 12-month Constant scores oftenotomy and tenodesis. The black diamonds represent the effect of individual studies, and thevertical lines show the corresponding 95% confidence intervals (CI). The size of the grey squaresreflects the weight of a particular study. The blue diamond reflects the overall or summary effect.The outer edges of the diamonds represent the CIs (PNG 92 KB)

Supplementary Fig. 4 A Forest plot that compares the level of pain on the Visual AnalogScale (VAS) of tenotomy and tenodesis at the 6-month follow-up. The black diamonds representthe effect of individual studies, and the vertical lines show the corresponding 95% confidenceintervals (CI). The size of the grey squares reflects the weight of a particular study. The bluediamond reflects the overall or summary effect. The outer edges of the diamonds represent theCIs (PNG 87 KB)

Supplementary Fig. 5 A Forest plot that compares the level of pain on the Visual Analog Scale (VAS) of tenotomy and tenodesis at the 12-month follow-up. The black diamonds represent the effect of individual studies, and the vertical lines show the corresponding 95% confidence intervals (CI). The size of the grey squares reflects the weight of a particular study. The blue diamond reflects the overall or summary effect. The outer edges of the diamonds represent theCIs (PNG 106 KB)

Supplementary Fig. 6 A Forest plot that compares the level of pain on the Visual AnalogScale (VAS) of tenotomy and tenodesis at the 24-month follow-up. The black diamonds representthe effect of individual studies, and the vertical lines show the corresponding 95% confidenceintervals (CI). The size of the grey squares reflects the weight of a particular study. The blue diamond reflects the overall or summary effect. The outer edges of the diamonds represent theCIs (PNG 105 KB)

Supplementary Fig. 7 A Forest plot that compares the number of bicipital cramping pain events of tenotomy and tenodesis, 6 months postoperatively. The black diamonds represent the effect of individual studies, and the vertical lines show the corresponding 95% confidence intervals (CI). The size of the grey squares reflects the weight of a particular study. The blue diamond reflects the overall or summary effect. The outer edges of the diamonds represent theCIs (PNG 108 KB)

Supplementary Fig. 8 A Forest plot that compares the operative time of tenotomy and tenodesis. The black diamonds represent the effect of individual studies, and the vertical lines show the corresponding 95% confidence intervals (CI). The size of the grey squares reflects the weight of a particular study. The blue diamond reflects the overall or summary effect. The outer edges of the diamonds represent the CIs (PNG 87 KB)

Supplementary Fig. 9 Trial sequential analysis (TSA) analysis for the 6-month elbowflexion strength in kg. The Z curve represents the studies of the meta-analysis in chronologicalorder. As the Z curve did not cross any boundaries, including the Alpha line, this outcome of themeta-analysis is inconclusive. More clinical trials are needed (PNG 75 KB)

Supplementary Fig. 10 Trial sequential analysis (TSA) analysis for the 12-month elbow flexion strength in kg. The Z curve represents the studies of the meta-analysis in chronological order. After the first study, the Z curve crossed the Conventional boundary, therefore the analysis was significant. However, the Z curve did not cross the Trial Sequential boundary, the analysis is therefore potentially spurious. The Z curve reached and crossed the Alpha line, thus the sample size exceeded the required meta-analysis sample size. This meta-analysis was inconclusive as there was potential spurious significance (p < 0.05). Since the required sample size was reached, further clinical trials are not required. Considering the raw data and comparing the TSA results to the forest plot, it is possible that some type of bias may influence these results (PNG 89 KB)

Supplementary Fig. 11 Trial sequential analysis (TSA) analysis for the 12-month forearmsupination strength in kg. The Z curve represents the studies of the meta-analysis in chronologicalorder. After the first study, the Z curve crossed the Conventional boundary. After the third study,the Z curve crossed the Trial Sequential boundary too, depicting that the analysis was trulysignificant from that point. The sample size did not exceed the required meta-analysis sample size(Alpha). This meta-analysis was inconclusive. More clinical trials are needed to confirm thesignificance (PNG 111 KB)

Supplementary Fig. 12 Trial sequential analysis (TSA) analysis for the 12-month Constant score. The Z curve represents the studies of the meta-analysis in chronological order. As the Z curve did not cross any boundaries, including the Alpha line, this outcome of the meta-analysis is inconclusive. More clinical trials are needed (PNG 85 KB)

Supplementary Fig. 13 Trial sequential analysis (TSA) analysis for the 3-month pain levels on the Visual Analog Scale (VAS) outcome. The Z curve represents the studies of the meta-analysis in chronological order. After the first study, the Z curve crossed the Conventional boundary. Therefore the analysis was significant. After the second study, the Z curve crossed the Trial sequential boundary showing real significance. The Z curve also reached and crossed theline of the Alpha after the second study. This means that the required sample size was reached after the second study. The tenotomy method was superior to the tenodesis method. Further clinical trials are not required (PNG 88 KB)

Supplementary Fig. 14 Trial sequential analysis (TSA) analysis for the 12-month pain levels on the Visual Analog Scale (VAS) outcome. The Z curve represents the studies of the meta-analysis in chronological order. As the Z curve did not cross any boundaries, including the Alpha line, the outcome of the meta-analysis is inconclusive. More clinical trials are needed (PNG 71 KB)

Supplementary Fig. 15 Trial sequential analysis (TSA) analysis for the occurrence of Popeye deformity at the 24-month follow-up. The Z curve represents the studies of the meta-analysis in chronological order. After the first study, the Z curve crossed the Conventional boundary, the Trial Sequential boundary, and the Alpha line. Therefore the analysis was truly significant from that point and reached the required sample size. The tenodesis method was superior to the tenotomy treatment. Further clinical trials are not required (PNG 83 KB)

Supplementary Fig. 16 Trial sequential analysis (TSA) analysis for the operative time in minutes. The Z curve represents the studies of the meta-analysis in chronological order. After the first study, the Z curve crossed the Conventional boundary as well as the Trial Sequential boundary. Therefore the analysis was potentially significant. However, after the second study, the Z curve crossed the Alpha line as well as the Futility boundary. This means that the sample size exceeded the required meta-analysis sample size (when the Z curve crossed the Alpha line). However, it also means that the significance of the meta-analysis was more spurious than reliable, as the Z curve crossed the Futility boundary. Therefore, this outcome of the meta-analysis was inconclusive since there was potential spurious significance (p < 0.05). As the required sample size was reached, further clinical trials are not required. Considering the raw data and comparing the TSA results to the forest plot, it is possible that some form of bias may have caused these results (PNG 89 KB)

Supplementary Fig. 17 Risk of bias assessment of the 6-month elbow flexion strengthoutcome, measured in kilogram (kg) (PNG 173 KB)

Supplementary Fig. 18 Risk of bias assessment of the 12-month elbow flexion strength outcome, measured in kilogram (kg) (PNG 177 KB)

Supplementary Fig. 19 Risk of bias assessment of the final elbow flexion strength outcome,measured in kilogram (kg) (PNG 190 KB)

Supplementary Fig. 20 Risk of bias assessment of the final elbow flexion strength outcome, measured in Strength Index (SI) (PNG 176 KB)

Supplementary Fig. 21 Risk of bias assessment of the 12-month forearm supination strength outcome, measured in kilogram (kg) (PNG 176 KB)

Supplementary Fig. 22 Risk of bias assessment of the final forearm supination strength outcome, measured in Strength Index (SI) (PNG 179 KB)

Supplementary Fig. 23 Risk of bias assessment of the 6-month Constant score outcome (PNG 169 KB)

Supplementary Fig. 24 Risk of bias assessment of the 12-month Constant score outcome (PNG 170 KB)

Supplementary Fig. 25 Risk of bias assessment of the final Constant score outcome (PNG 179 KB)

Supplementary Fig. 26 Risk of bias assessment of the final American Shoulder and Elbow Surgeons (ASES) score outcome (PNG 160 KB)

Supplementary Fig. 27 Risk of bias assessment of the final Simple Shoulder Test (SST) score outcome (PNG 168 KB)

Supplementary Fig. 28 Risk of bias assessment of the 3-month pain levels on the Visual Analog scale (VAS) outcome (PNG 168 KB)

Supplementary Fig. 29 Risk of bias assessment of the 6-month pain levels on the Visual Analog scale (VAS) outcome (PNG 180 KB)

Supplementary Fig. 30 Risk of bias assessment of the 12-month pain levels on the Visual Analog scale (VAS) outcome (PNG 179 KB)

Supplementary Fig. 31 Risk of bias assessment of the 24-month pain levels on the Visual Analog scale (VAS) outcome (PNG 180 KB)

Supplementary Fig. 32 Risk of bias assessment of the final pain levels on the Visual Analog scale (VAS) outcome (PNG 193 KB)

Supplementary Fig. 33 Risk of bias assessment of the number of bicipital cramping pain events at 6 months outcome (PNG 163 KB)

Supplementary Fig. 34 Risk of bias assessment of the number of bicipital cramping pain events at the final evaluation outcome (PNG 195 KB)

Supplementary Fig. 35 Risk of bias assessment of the number of bicipital groove pain events at the final evaluation outcome (PNG 168 KB)

Supplementary Fig. 36 Risk of bias assessment of the number of Popeye deformity events at 24 months outcome (PNG 190 KB)

Supplementary Fig. 37 Risk of bias assessment of the number of Popeye deformity events at the final evaluation outcome (PNG 207 KB)

Supplementary Fig. 38 Risk of bias assessment of the operative time outcome measured inminutes (PNG 180 KB)

## Data Availability

Data and material is available from the first author. Email: v.matyasvajda@gmail.com.
