# Supplementary material for: Tenodesis yields better functional results than tenotomy in long head of the biceps tendon operations—a systematic review and meta-analysis
Source: Int Orthop. 2022 Mar 7;46(5):1037–51. doi: 10.1007/s00264-022-05338-9 (PMC9001564; doi:10.1007/s00264-022-05338-9)
Supplement: Supplementary file 1 — Supplementary Table 1 Summary of the certainty of evidence according to the GRADE (DOCX 21 KB) [file 264_2022_5338_MOESM1_ESM.docx]

**Supplementary table 1: Summary of the certainty of evidence according to the GRADE analysis**

|  | **Certainty assessment** | | | | | | **№ of patients** | | **Effect** | | **Certainty** | **Importance** |
| --- | --- | --- | --- | --- | --- | --- | --- | --- | --- | --- | --- | --- |
| Outcome | **№ of studies** | **Risk of bias** | **Inconsistency** | **Indirectness** | **Imprecision** | **Other considerations** | **tenotomy** | **tenodesis** | **Relative** | **Absolute** |  |  |
|  |  |  |  |  |  |  |  |  | **(95% CI)** | **(95% CI)** |  |  |
| Elbow flexion strength in kg, 6-month scores | 3 | not serious | serious ^a^ | not serious | serious ^b^ | none | 80 | 80 | - | WMD: 2.82 | ⨁⨁◯◯ | IMPORTANT |
|  |  |  |  |  |  |  |  |  |  | (-1.79 to 7.22) | LOW |  |
| Elbow flexion strength in kg, 12-month scores | 3 | not serious | not serious | serious ^c^ | not serious | none | 80 | 74 | - | WMD: 3.67 | ⨁⨁⨁◯ | IMPORTANT |
|  |  |  |  |  |  |  |  |  |  | (1.07 to 6.27) | MODERATE |  |
| Supination strength in kg, 12-month scores | 3 | not serious | not serious | serious ^c^ | serious ^b^ | none | 80 | 69 | - | WMD: 0.36 | ⨁⨁◯◯ | IMPORTANT |
|  |  |  |  |  |  |  |  |  |  | (0.08 to 0.64) | LOW |  |
| Constant score, 6-month scores | 3 | not serious | not serious | not serious | serious ^d^ | none | 71 | 66 | - | WMD: 0.78 | ⨁⨁⨁◯ | IMPORTANT |
|  |  |  |  |  |  |  |  |  |  | (-2.44 to 4.00) | MODERATE |  |
| Constant score, 12-month scores | 3 | not serious | not serious | serious ^c^ | serious ^b^ | none | 63 | 60 | - | WMD: 2.26 | ⨁⨁◯◯ | IMPORTANT |
|  |  |  |  |  |  |  |  |  |  | (-1.12 to 5.65) | LOW |  |
| Pain on VAS, 3-month scores | 3 | not serious | not serious | not serious | not serious | none | 84 | 84 | - | WMD: 0.99 | ⨁⨁⨁⨁ | IMPORTANT |
|  |  |  |  |  |  |  |  |  |  | (0.51 to 1.48) | HIGH |  |
| Pain on VAS, 6-month scores | 4 | not serious | not serious | not serious | serious ^d^ | none | 124 | 126 | - | WMD: 0.05 | ⨁⨁⨁◯ | IMPORTANT |
|  |  |  |  |  |  |  |  |  |  | (-0.21 to 0.30) | MODERATE |  |
| Pain on VAS, 12-month scores | 4 | not serious | serious ^e^ | serious ^c^ | serious ^b^ | none | 116 | 115 | - | WMD: 0.19 | ⨁◯◯◯ | IMPORTANT |
|  |  |  |  |  |  |  |  |  |  | (-0.26 to 0.63) | VERY LOW |  |
| Pain on VAS, 24-month scores | 4 | not serious | not serious | not serious | serious ^d^ | none | 132 | 119 | - | WMD: 0.01 | ⨁⨁⨁◯ | IMPORTANT |
|  |  |  |  |  |  |  |  |  |  | (-0.04 to 0.07) | MODERATE |  |
| 6-month bicipital cramping pain events | 3 | not serious | not serious | not serious | serious ^d^ | none | 4/69 (5.8%) | 4/64 (6.3%) | OR: 0.92 | 5 fewer per 1 000 | ⨁⨁⨁◯ | IMPORTANT |
|  |  |  |  |  |  |  |  |  | (0.09 to 9.07) | (from 57 fewer to 314 more) | MODERATE |  |
| 24-month Popeye deformity events | 3 | serious ^f^ | not serious | not serious | not serious | none | 40/105 (38.1%) | 10/97 (10.3%) | OR: 0.19 | 82 fewer per 1 000 | ⨁⨁⨁◯ | IMPORTANT |
|  |  |  |  |  |  |  |  |  | (0.08 to 0.41) | (from 94 fewer to 58 fewer) | MODERATE |  |
| Operative time | 3 | serious ^f^ | serious ^g^ | not serious | serious ^h^ | none | 152 | 150 | - | WMD: 17.15 | ⨁◯◯◯ | NOT IMPORTANT |
|  |  |  |  |  |  |  |  |  |  | (-2.05 to 36.35) | VERY LOW |  |

CI: Confidence Interval; OR: Odds Ratio, WMD: Weighted Mean Difference, kg: kilogram, VAS: Visual Analog Scale, **a:** Significant heterogeneity was detected (I square test: 71.7%, p=0.029), **b:** According to our Trial Sequential Analysis more clinical trials are needed, **c:** One of the studies only include men, **d:** Small sample size, wide CI, **e:** Significant heterogeneity was detected (I square test: 80.1%, p=0.002), **f:** According to our Risk of Bias assessment, one of the studies carries high risk of bias, **g:** Significant heterogeneity was detected (I square test: 97.5%, p<0.001), **h:** According to our Trial Sequential Analysis, the analysis of this outcome was inconclusive as there was potential spurious significance (p < 0.05)
