# Supplementary figures and images for: Tenodesis yields better functional results than tenotomy in long head of the biceps tendon operations—a systematic review and meta-analysis
Source: Int Orthop. 2022 Mar 7;46(5):1037–51. doi: 10.1007/s00264-022-05338-9 (PMC9001564; doi:10.1007/s00264-022-05338-9)

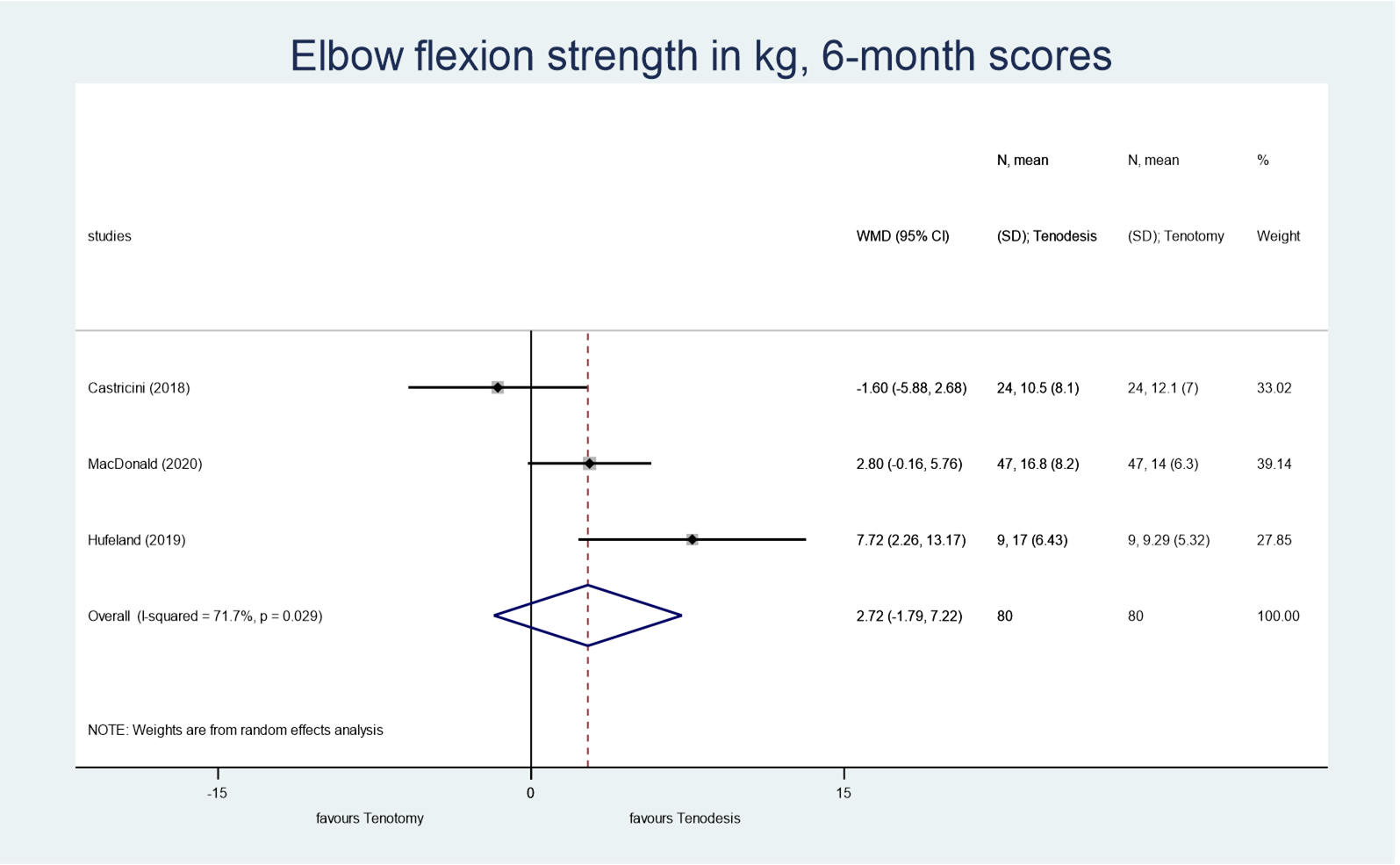

Supplement: Supplementary file 2 — Supplementary Fig. 1 A Forest plot that compares the results of elbow flexion strengthmeasurements in kilogram (kg) in tenotomy and tenodesis 6 months postoperatively. The blackdiamonds represent the effect of individual studies, and the vertical lines show the corresponding95% confidence intervals (CI). The size of the grey squares reflects the weight of a particularstudy. The blue diamond reflects the overall or summary effect. The outer edges of the diamondsrepresent the CIs (PNG 100 KB) [file 264_2022_5338_MOESM2_ESM.png]

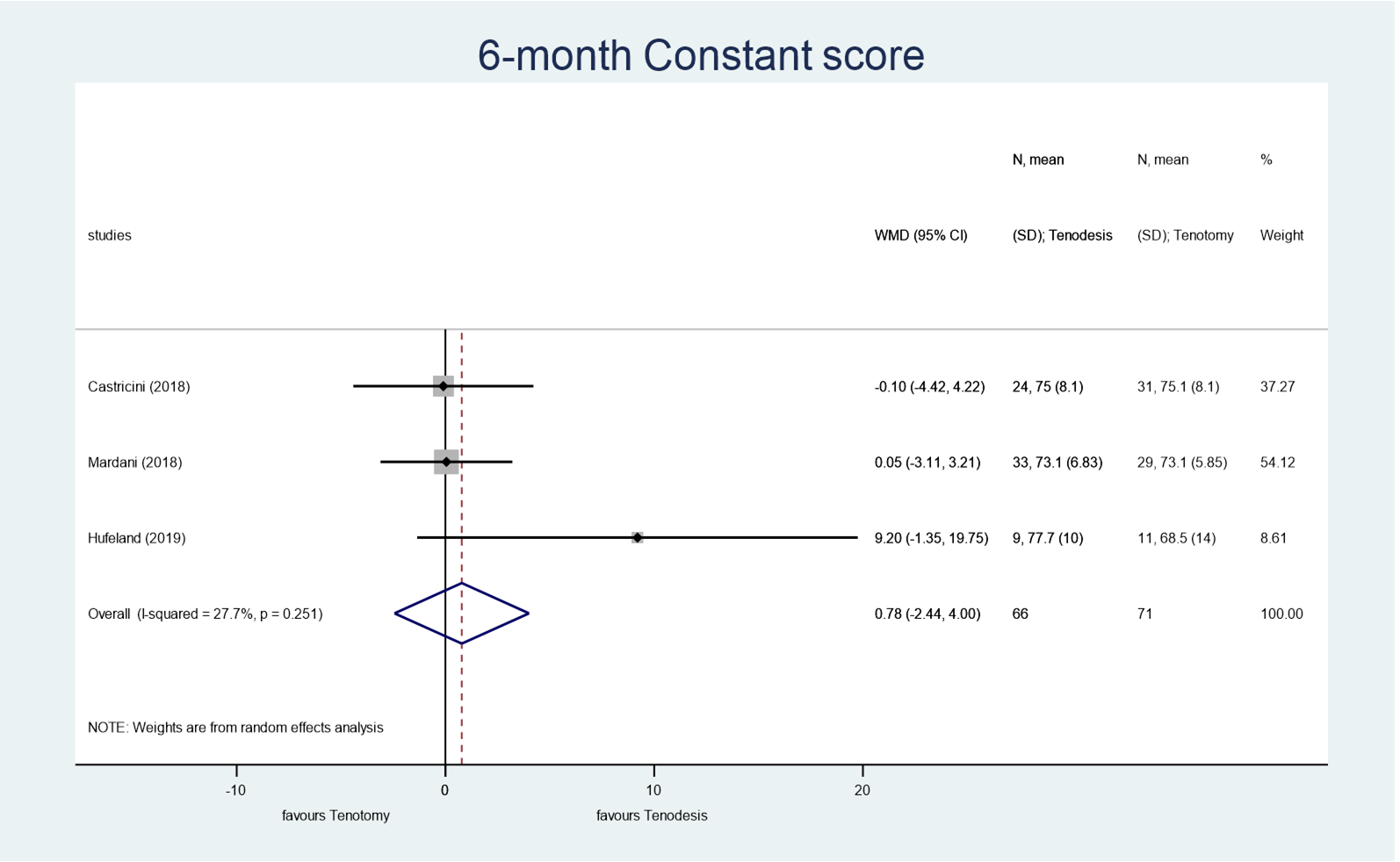

Supplement: Supplementary file 3 — Supplementary Fig. 2 A Forest plot that compares the 6-month Constant scores of tenotomyand tenodesis. The black diamonds represent the effect of individual studies, and the vertical linesshow the corresponding 95% confidence intervals (CI). The size of the grey squares reflects theweight of a particular study. The blue diamond reflects the overall or summary effect. The outeredges of the diamonds represent the CIs (PNG 94 KB) [file 264_2022_5338_MOESM3_ESM.png]

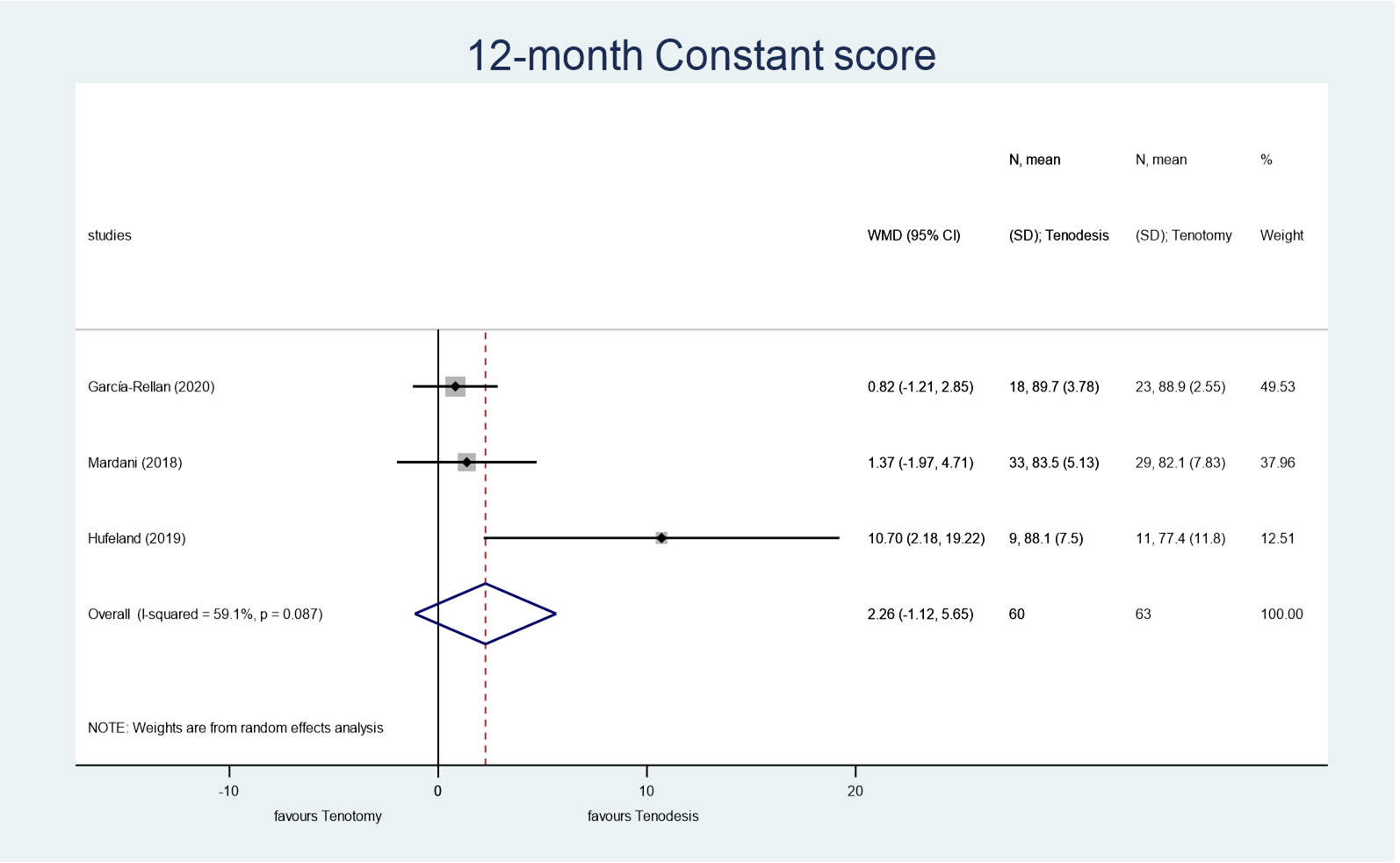

Supplement: Supplementary file 4 — Supplementary Fig. 3 A Forest plot that compares the 12-month Constant scores oftenotomy and tenodesis. The black diamonds represent the effect of individual studies, and thevertical lines show the corresponding 95% confidence intervals (CI). The size of the grey squaresreflects the weight of a particular study. The blue diamond reflects the overall or summary effect.The outer edges of the diamonds represent the CIs (PNG 92 KB) [file 264_2022_5338_MOESM4_ESM.png]

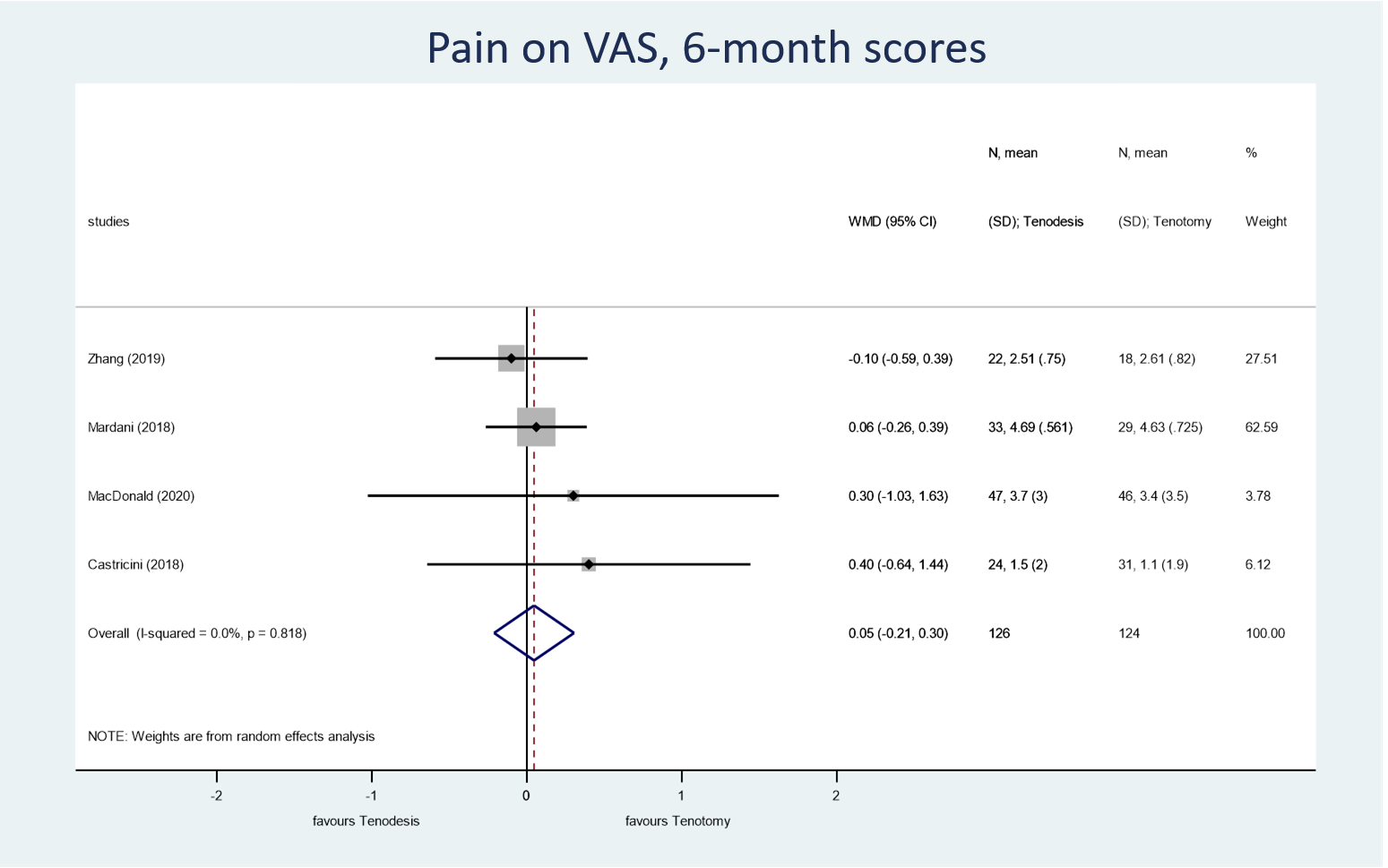

Supplement: Supplementary file 5 — Supplementary Fig. 4 A Forest plot that compares the level of pain on the Visual AnalogScale (VAS) of tenotomy and tenodesis at the 6-month follow-up. The black diamonds representthe effect of individual studies, and the vertical lines show the corresponding 95% confidenceintervals (CI). The size of the grey squares reflects the weight of a particular study. The bluediamond reflects the overall or summary effect. The outer edges of the diamonds represent theCIs (PNG 87 KB) [file 264_2022_5338_MOESM5_ESM.png]

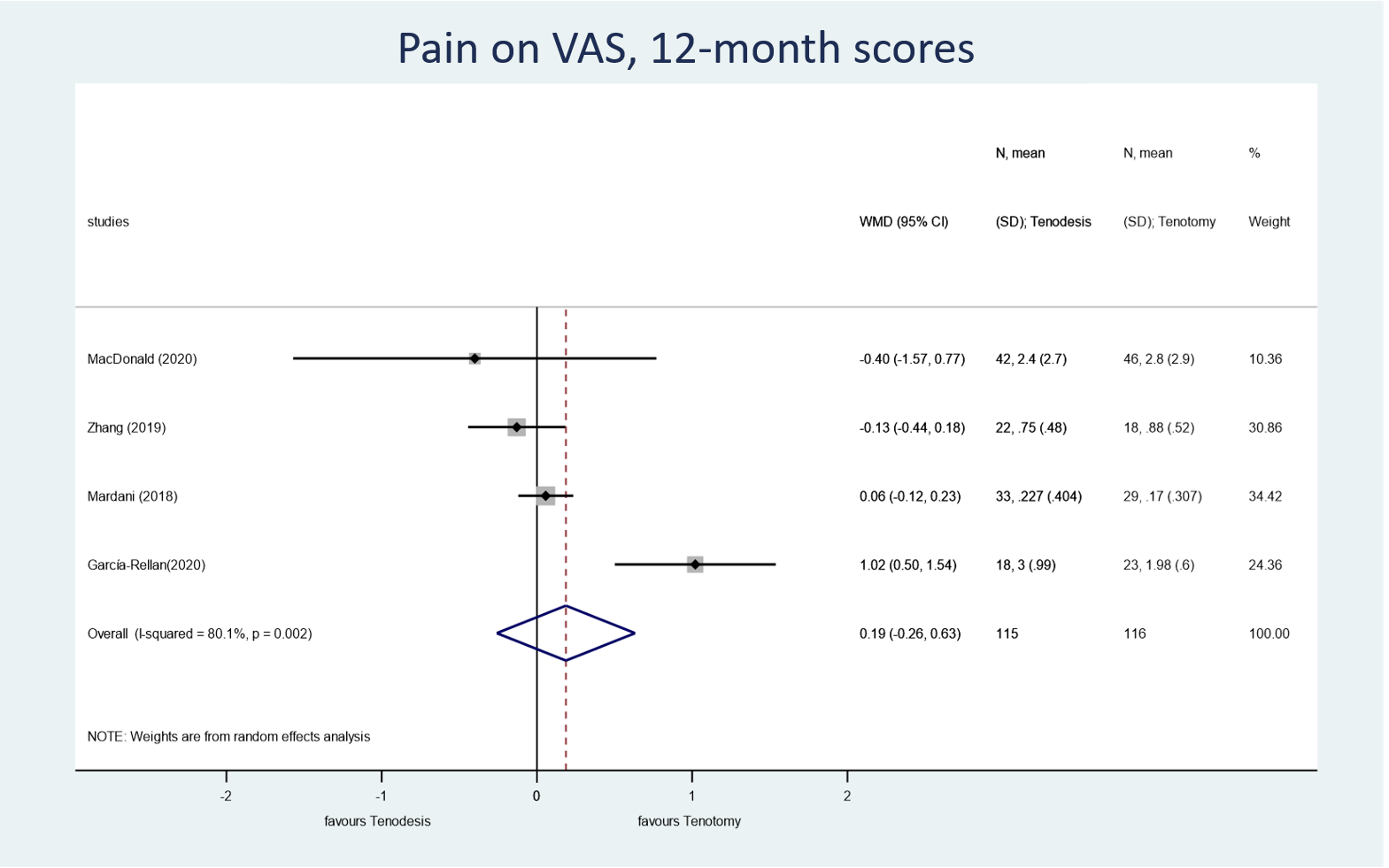

Supplement: Supplementary file 6 — Supplementary Fig. 5 A Forest plot that compares the level of pain on the Visual Analog Scale (VAS) of tenotomy and tenodesis at the 12-month follow-up. The black diamonds represent the effect of individual studies, and the vertical lines show the corresponding 95% confidence intervals (CI). The size of the grey squares reflects the weight of a particular study. The blue diamond reflects the overall or summary effect. The outer edges of the diamonds represent theCIs (PNG 106 KB) [file 264_2022_5338_MOESM6_ESM.png]

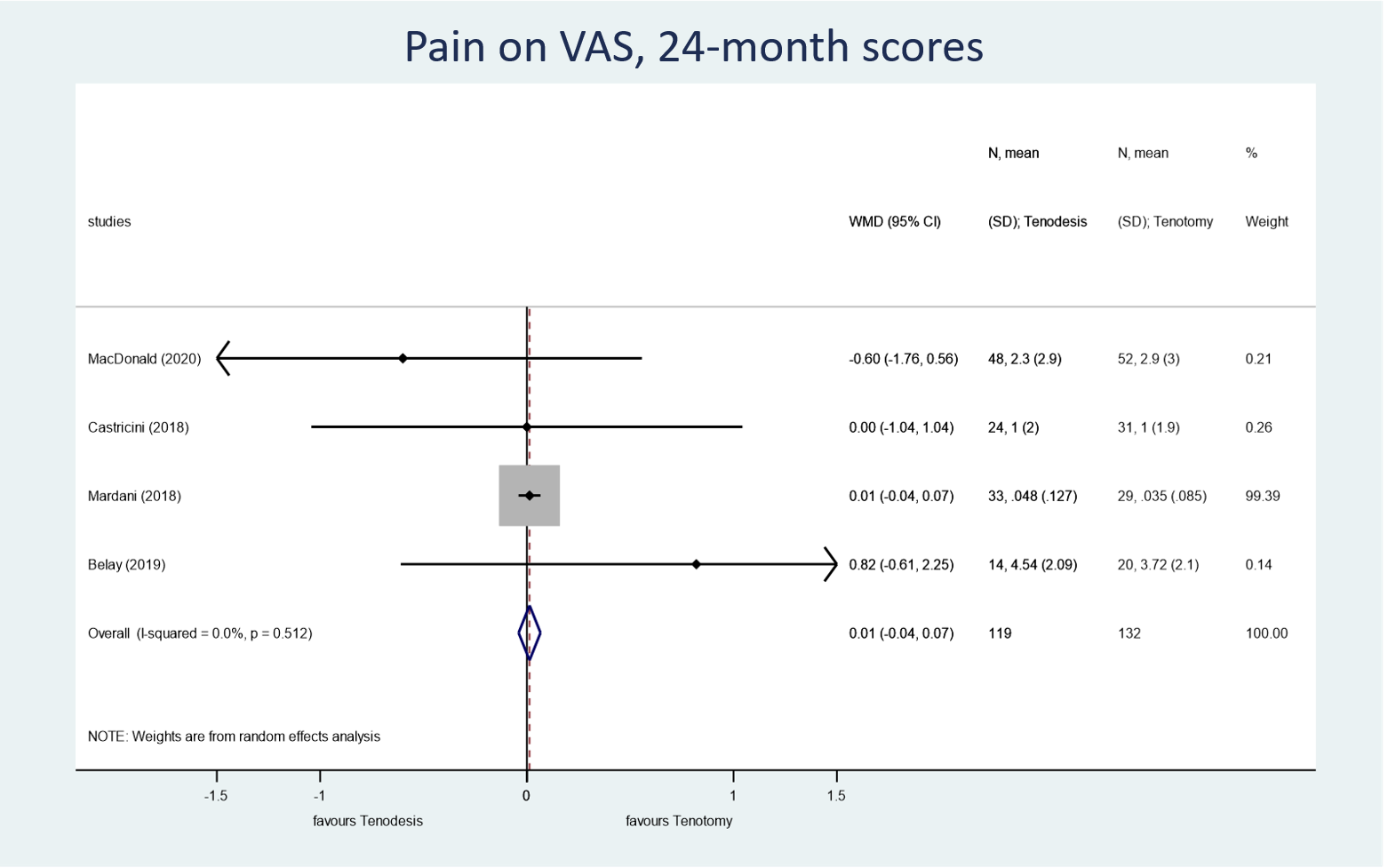

Supplement: Supplementary file 7 — Supplementary Fig. 6 A Forest plot that compares the level of pain on the Visual AnalogScale (VAS) of tenotomy and tenodesis at the 24-month follow-up. The black diamonds representthe effect of individual studies, and the vertical lines show the corresponding 95% confidenceintervals (CI). The size of the grey squares reflects the weight of a particular study. The blue diamond reflects the overall or summary effect. The outer edges of the diamonds represent theCIs (PNG 105 KB) [file 264_2022_5338_MOESM7_ESM.png]

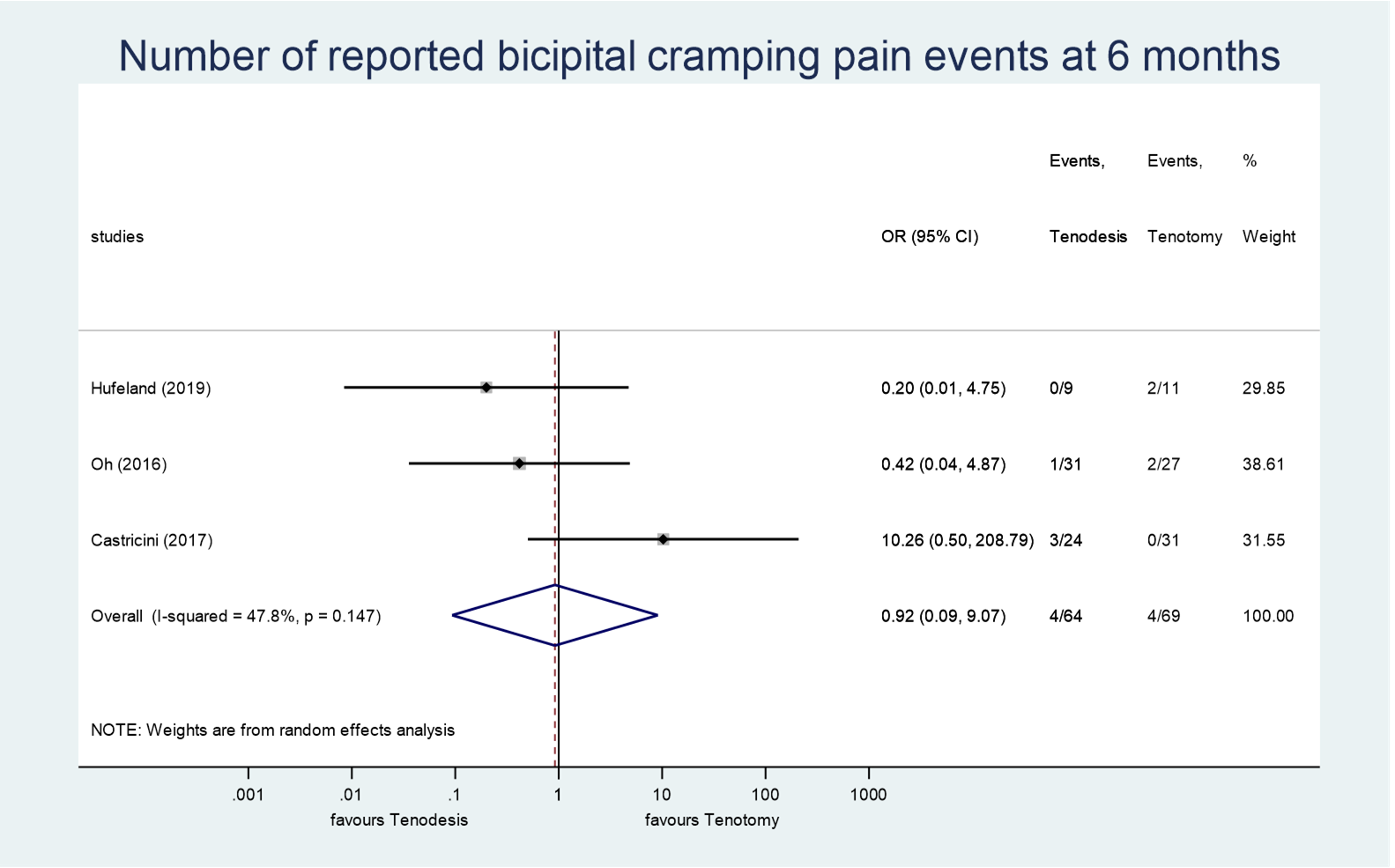

Supplement: Supplementary file 8 — Supplementary Fig. 7 A Forest plot that compares the number of bicipital cramping pain events of tenotomy and tenodesis, 6 months postoperatively. The black diamonds represent the effect of individual studies, and the vertical lines show the corresponding 95% confidence intervals (CI). The size of the grey squares reflects the weight of a particular study. The blue diamond reflects the overall or summary effect. The outer edges of the diamonds represent theCIs (PNG 108 KB) [file 264_2022_5338_MOESM8_ESM.png]

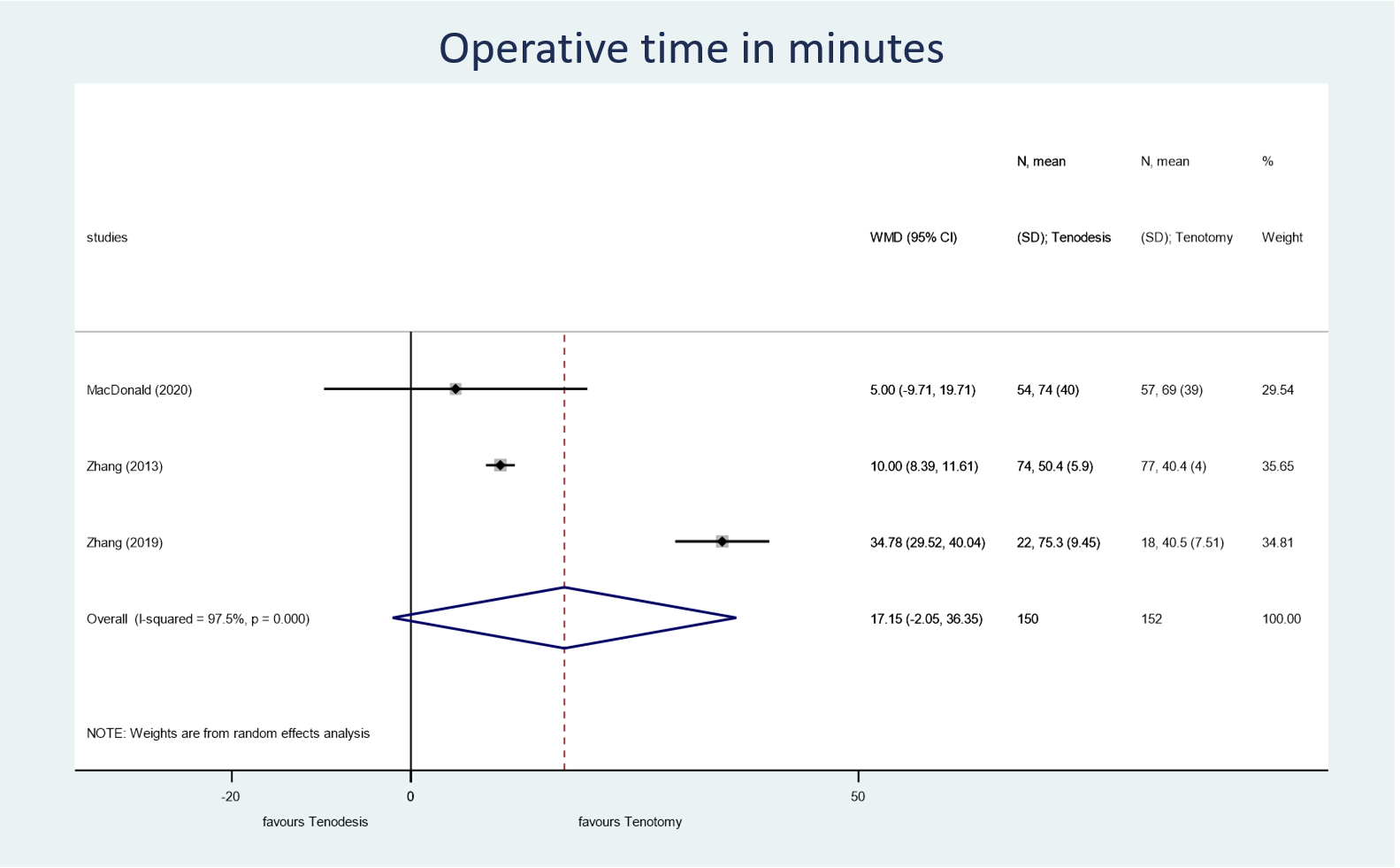

Supplement: Supplementary file 9 — Supplementary Fig. 8 A Forest plot that compares the operative time of tenotomy and tenodesis. The black diamonds represent the effect of individual studies, and the vertical lines show the corresponding 95% confidence intervals (CI). The size of the grey squares reflects the weight of a particular study. The blue diamond reflects the overall or summary effect. The outer edges of the diamonds represent the CIs (PNG 87 KB) [file 264_2022_5338_MOESM9_ESM.png]

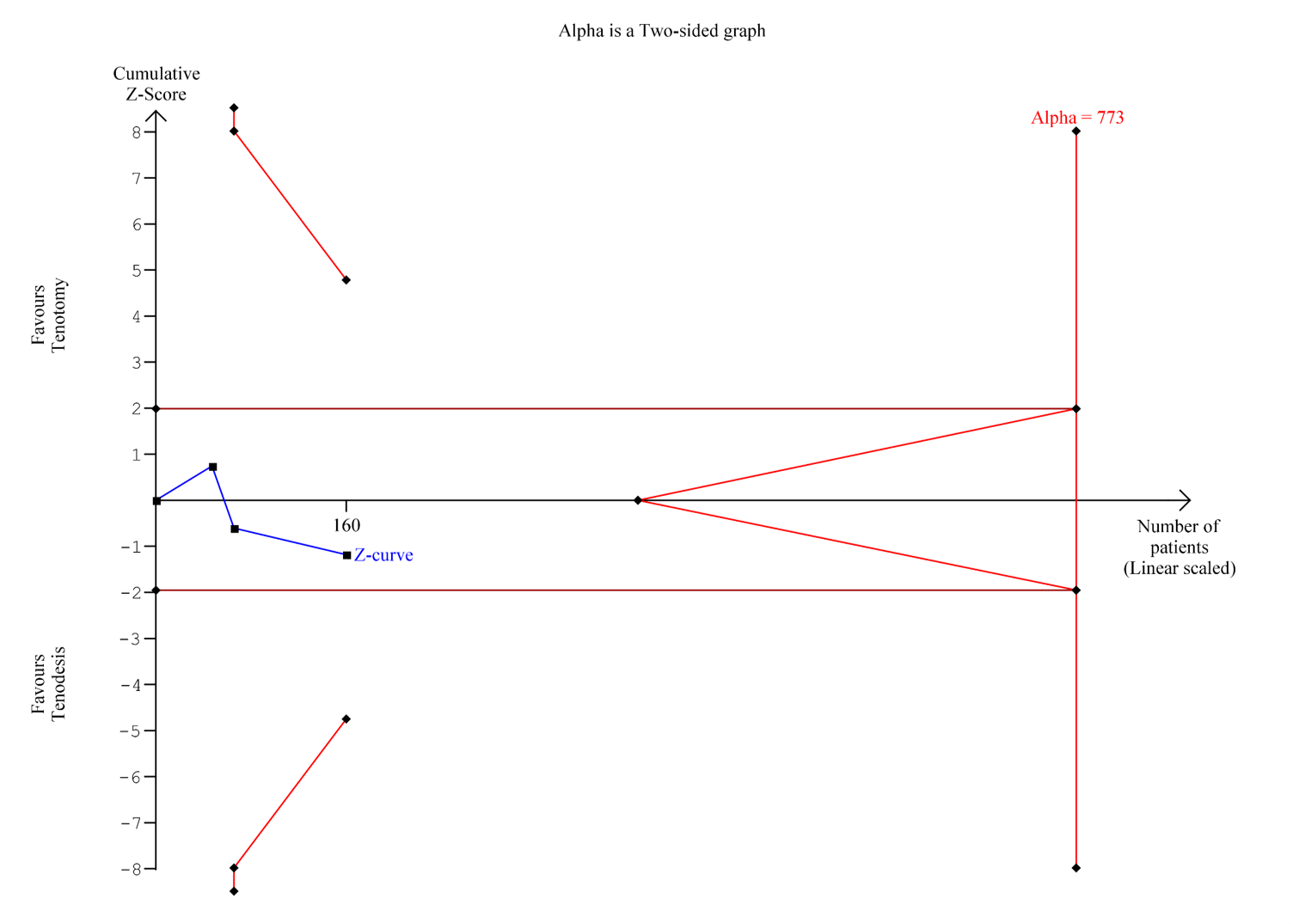

Supplement: Supplementary file 10 — Supplementary Fig. 9 Trial sequential analysis (TSA) analysis for the 6-month elbowflexion strength in kg. The Z curve represents the studies of the meta-analysis in chronologicalorder. As the Z curve did not cross any boundaries, including the Alpha line, this outcome of themeta-analysis is inconclusive. More clinical trials are needed (PNG 75 KB) [file 264_2022_5338_MOESM10_ESM.png]

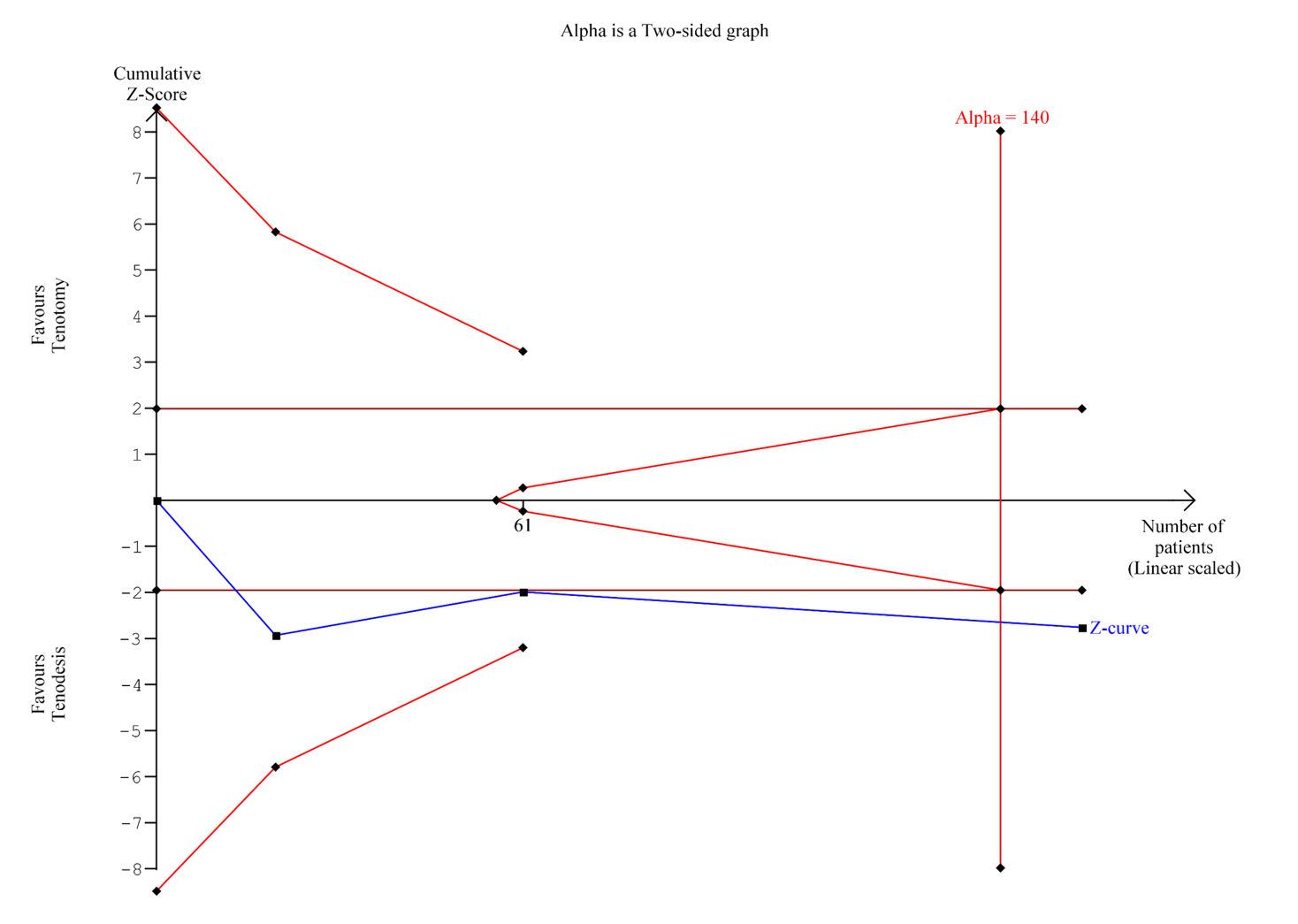

Supplement: Supplementary file 11 — Supplementary Fig. 10 Trial sequential analysis (TSA) analysis for the 12-month elbow flexion strength in kg. The Z curve represents the studies of the meta-analysis in chronological order. After the first study, the Z curve crossed the Conventional boundary, therefore the analysis was significant. However, the Z curve did not cross the Trial Sequential boundary, the analysis is therefore potentially spurious. The Z curve reached and crossed the Alpha line, thus the sample size exceeded the required meta-analysis sample size. This meta-analysis was inconclusive as there was potential spurious significance (p < 0.05). Since the required sample size was reached, further clinical trials are not required. Considering the raw data and comparing the TSA results to the forest plot, it is possible that some type of bias may influence these results (PNG 89 KB) [file 264_2022_5338_MOESM11_ESM.png]

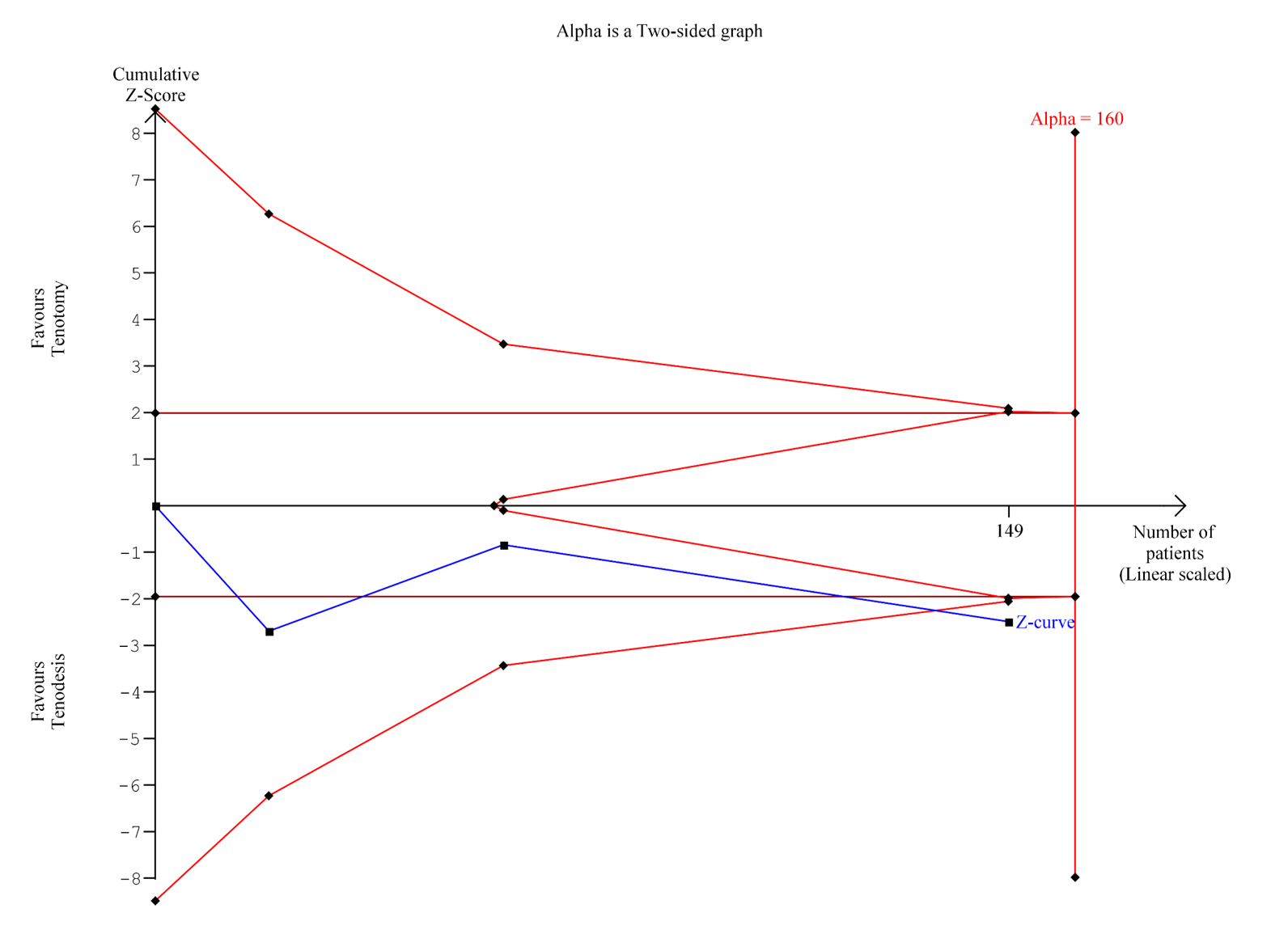

Supplement: Supplementary file 12 — Supplementary Fig. 11 Trial sequential analysis (TSA) analysis for the 12-month forearmsupination strength in kg. The Z curve represents the studies of the meta-analysis in chronologicalorder. After the first study, the Z curve crossed the Conventional boundary. After the third study,the Z curve crossed the Trial Sequential boundary too, depicting that the analysis was trulysignificant from that point. The sample size did not exceed the required meta-analysis sample size(Alpha). This meta-analysis was inconclusive. More clinical trials are needed to confirm thesignificance (PNG 111 KB) [file 264_2022_5338_MOESM12_ESM.png]

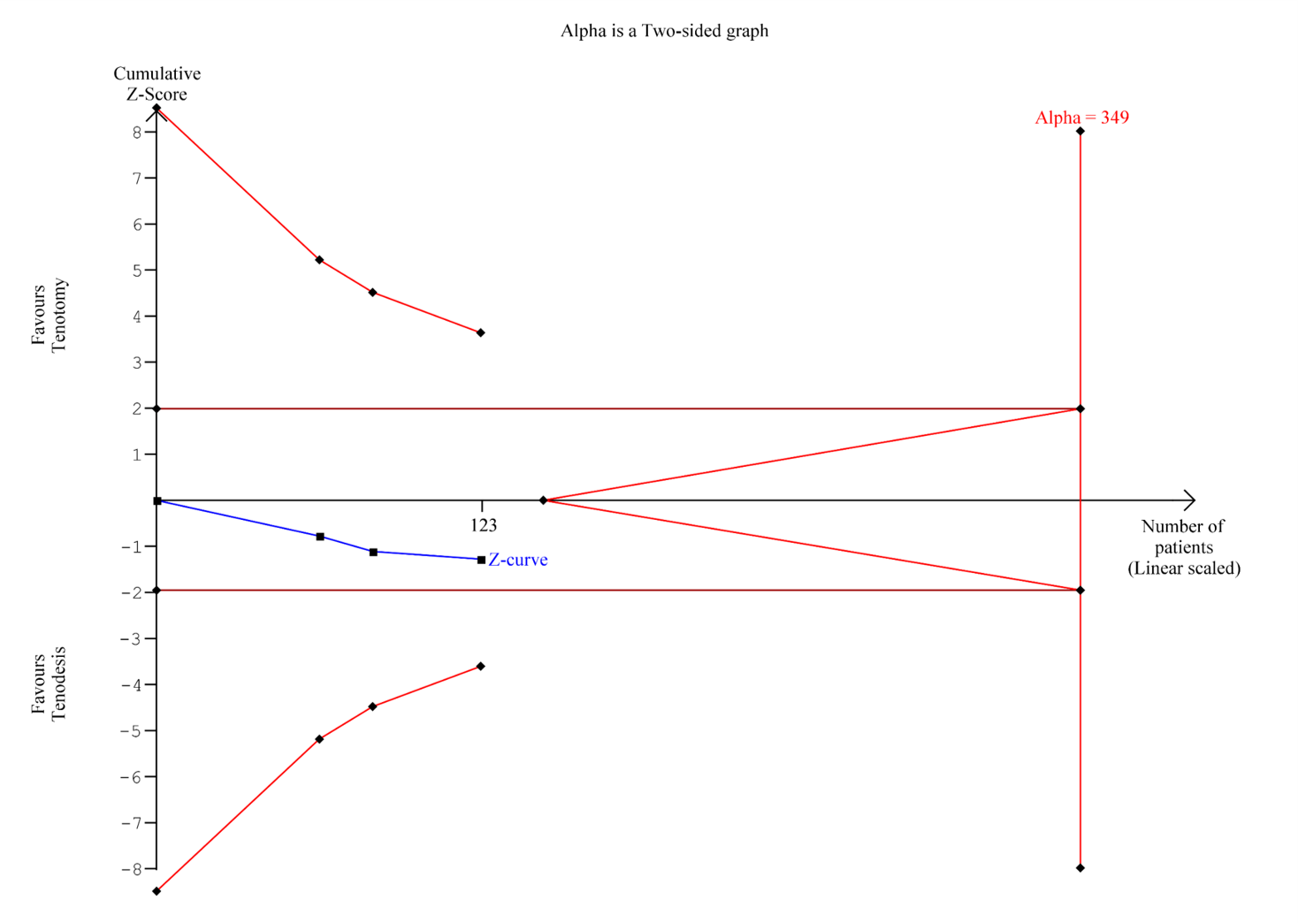

Supplement: Supplementary file 13 — Supplementary Fig. 12 Trial sequential analysis (TSA) analysis for the 12-month Constant score. The Z curve represents the studies of the meta-analysis in chronological order. As the Z curve did not cross any boundaries, including the Alpha line, this outcome of the meta-analysis is inconclusive. More clinical trials are needed (PNG 85 KB) [file 264_2022_5338_MOESM13_ESM.png]

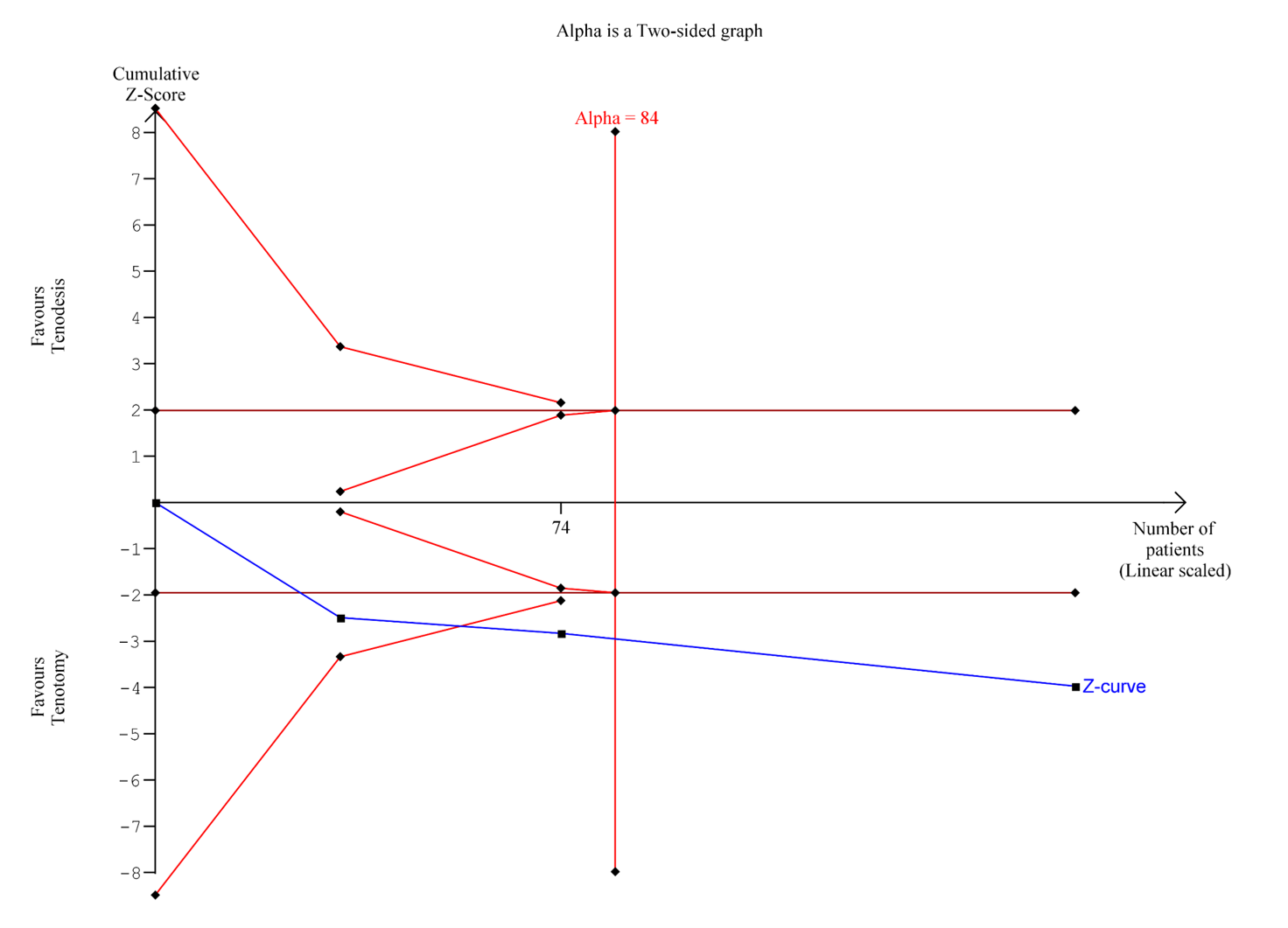

Supplement: Supplementary file 14 — Supplementary Fig. 13 Trial sequential analysis (TSA) analysis for the 3-month pain levels on the Visual Analog Scale (VAS) outcome. The Z curve represents the studies of the meta-analysis in chronological order. After the first study, the Z curve crossed the Conventional boundary. Therefore the analysis was significant. After the second study, the Z curve crossed the Trial sequential boundary showing real significance. The Z curve also reached and crossed theline of the Alpha after the second study. This means that the required sample size was reached after the second study. The tenotomy method was superior to the tenodesis method. Further clinical trials are not required (PNG 88 KB) [file 264_2022_5338_MOESM14_ESM.png]

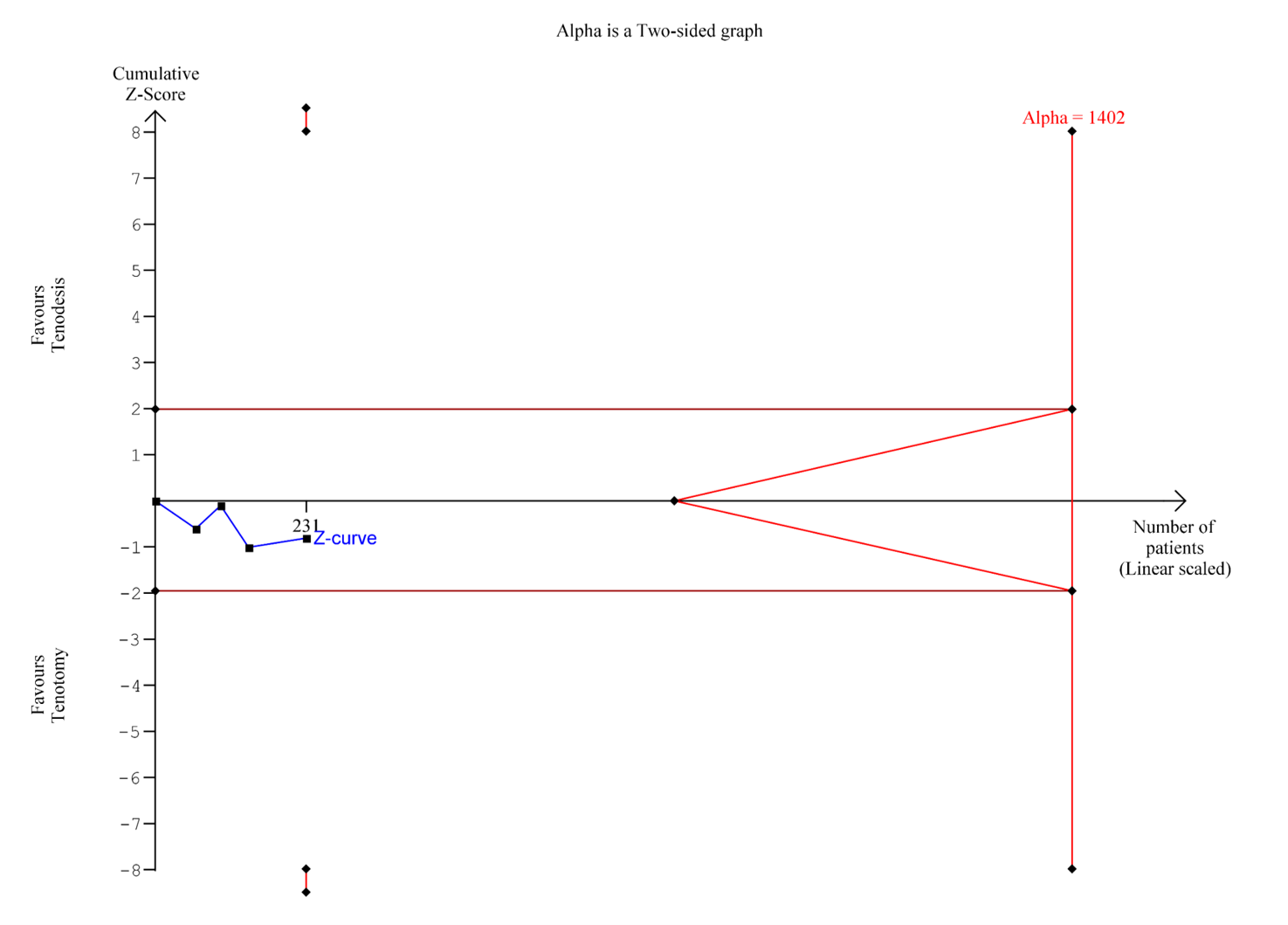

Supplement: Supplementary file 15 — Supplementary Fig. 14 Trial sequential analysis (TSA) analysis for the 12-month pain levels on the Visual Analog Scale (VAS) outcome. The Z curve represents the studies of the meta-analysis in chronological order. As the Z curve did not cross any boundaries, including the Alpha line, the outcome of the meta-analysis is inconclusive. More clinical trials are needed (PNG 71 KB) [file 264_2022_5338_MOESM15_ESM.png]

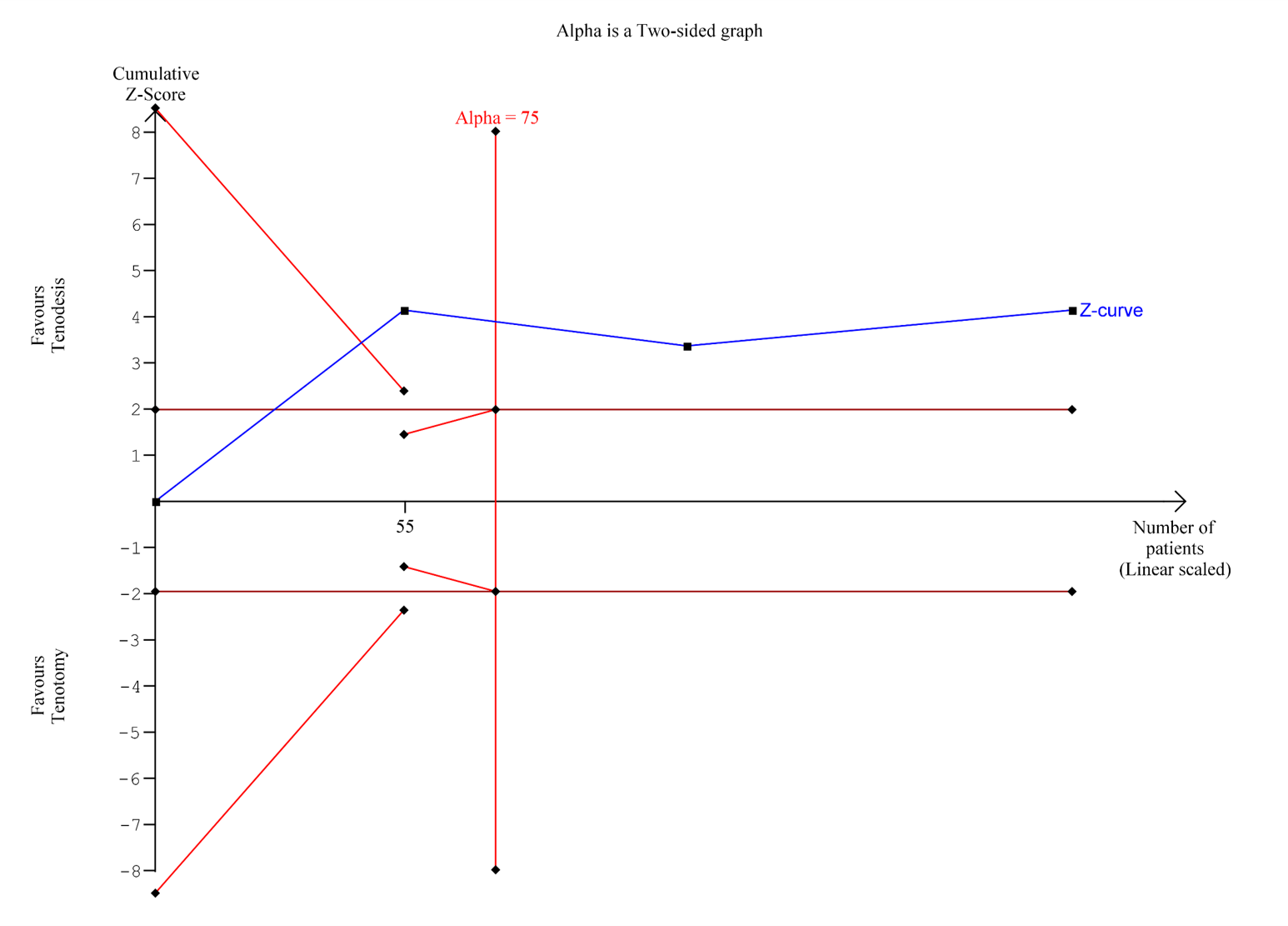

Supplement: Supplementary file 16 — Supplementary Fig. 15 Trial sequential analysis (TSA) analysis for the occurrence of Popeye deformity at the 24-month follow-up. The Z curve represents the studies of the meta-analysis in chronological order. After the first study, the Z curve crossed the Conventional boundary, the Trial Sequential boundary, and the Alpha line. Therefore the analysis was truly significant from that point and reached the required sample size. The tenodesis method was superior to the tenotomy treatment. Further clinical trials are not required (PNG 83 KB) [file 264_2022_5338_MOESM16_ESM.png]

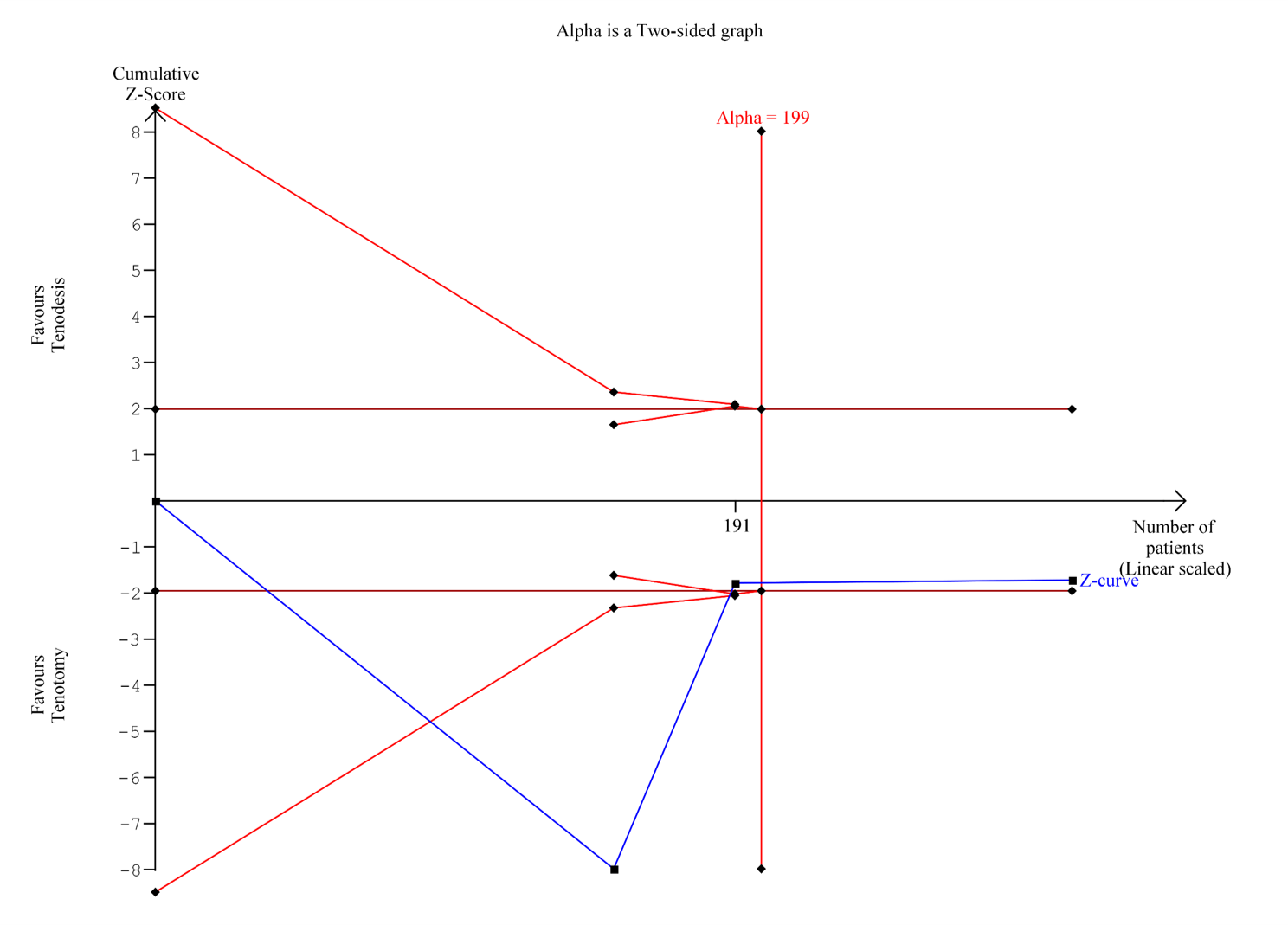

Supplement: Supplementary file 17 — Supplementary Fig. 16 Trial sequential analysis (TSA) analysis for the operative time in minutes. The Z curve represents the studies of the meta-analysis in chronological order. After the first study, the Z curve crossed the Conventional boundary as well as the Trial Sequential boundary. Therefore the analysis was potentially significant. However, after the second study, the Z curve crossed the Alpha line as well as the Futility boundary. This means that the sample size exceeded the required meta-analysis sample size (when the Z curve crossed the Alpha line). However, it also means that the significance of the meta-analysis was more spurious than reliable, as the Z curve crossed the Futility boundary. Therefore, this outcome of the meta-analysis was inconclusive since there was potential spurious significance (p < 0.05). As the required sample size was reached, further clinical trials are not required. Considering the raw data and comparing the TSA results to the forest plot, it is possible that some form of bias may have caused these results (PNG 89 KB) [file 264_2022_5338_MOESM17_ESM.png]

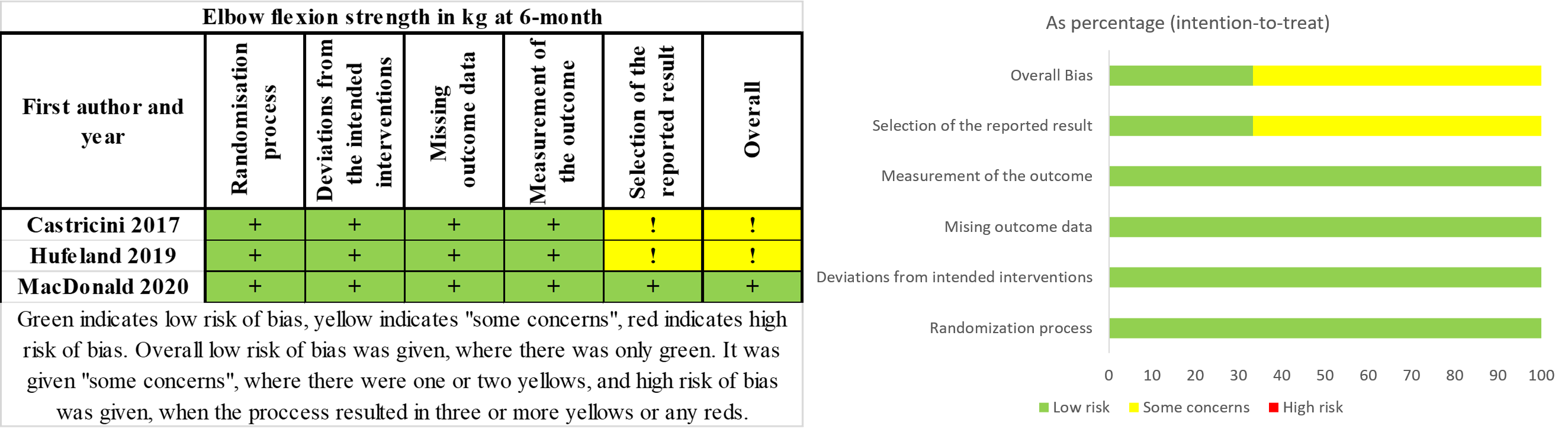

Supplement: Supplementary file 18 — Supplementary Fig. 17 Risk of bias assessment of the 6-month elbow flexion strengthoutcome, measured in kilogram (kg) (PNG 173 KB) [file 264_2022_5338_MOESM18_ESM.png]

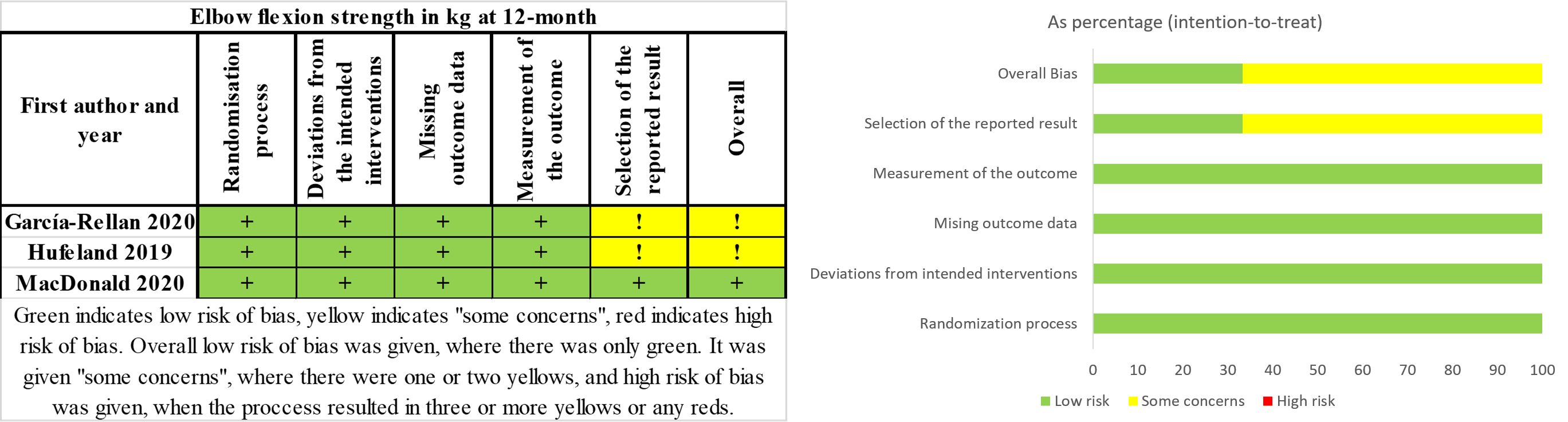

Supplement: Supplementary file 19 — Supplementary Fig. 18 Risk of bias assessment of the 12-month elbow flexion strength outcome, measured in kilogram (kg) (PNG 177 KB) [file 264_2022_5338_MOESM19_ESM.png]

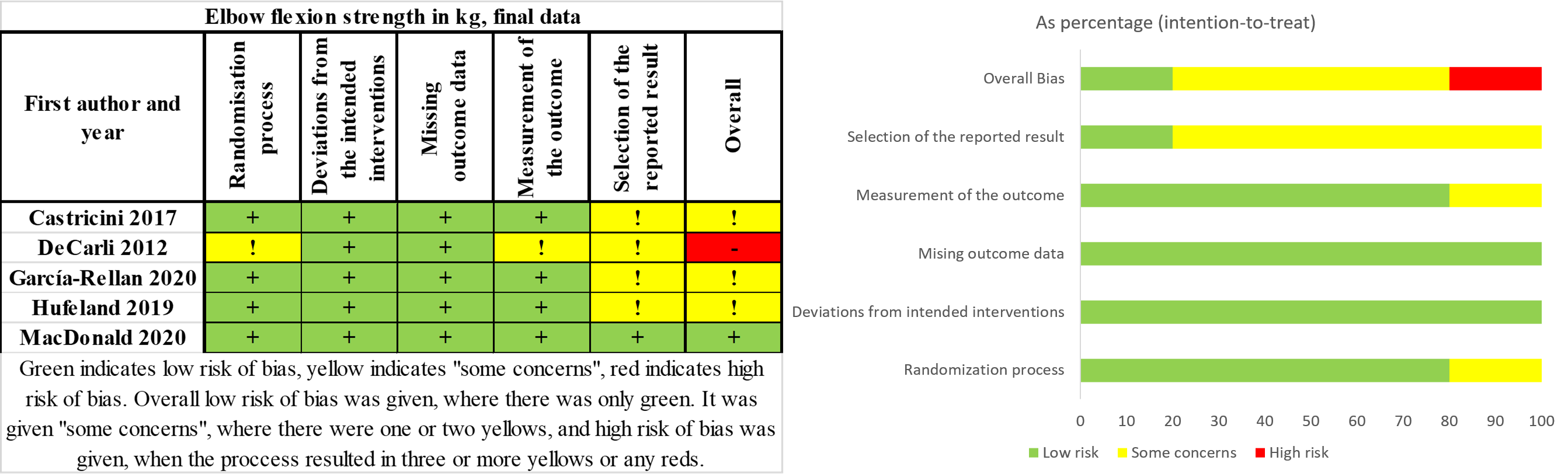

Supplement: Supplementary file 20 — Supplementary Fig. 19 Risk of bias assessment of the final elbow flexion strength outcome,measured in kilogram (kg) (PNG 190 KB) [file 264_2022_5338_MOESM20_ESM.png]

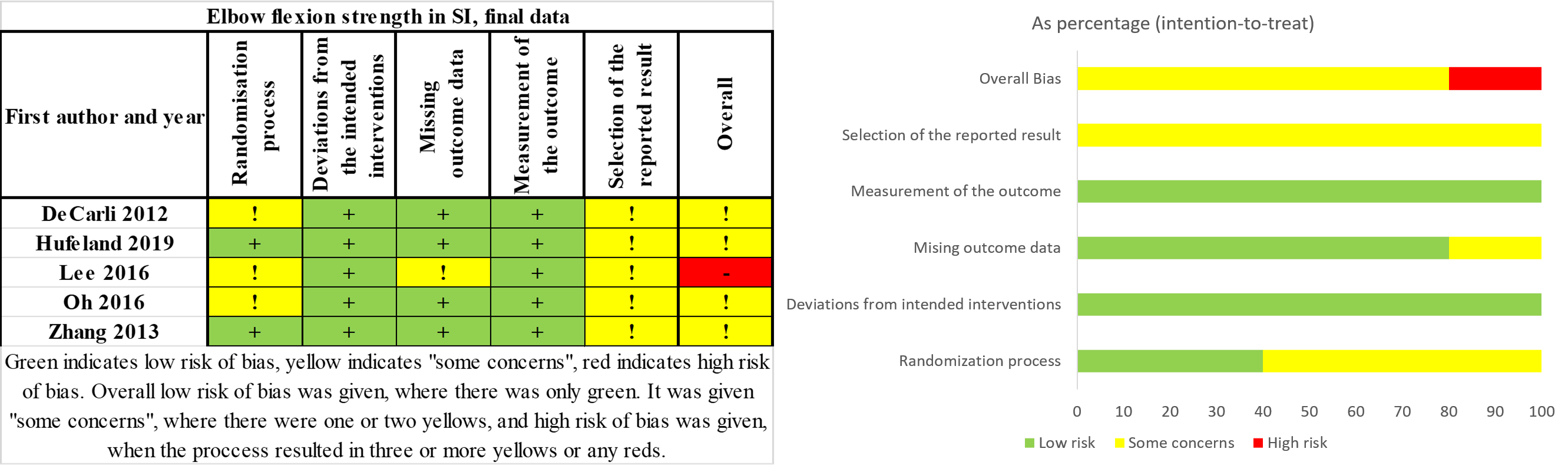

Supplement: Supplementary file 21 — Supplementary Fig. 20 Risk of bias assessment of the final elbow flexion strength outcome, measured in Strength Index (SI) (PNG 176 KB) [file 264_2022_5338_MOESM21_ESM.png]

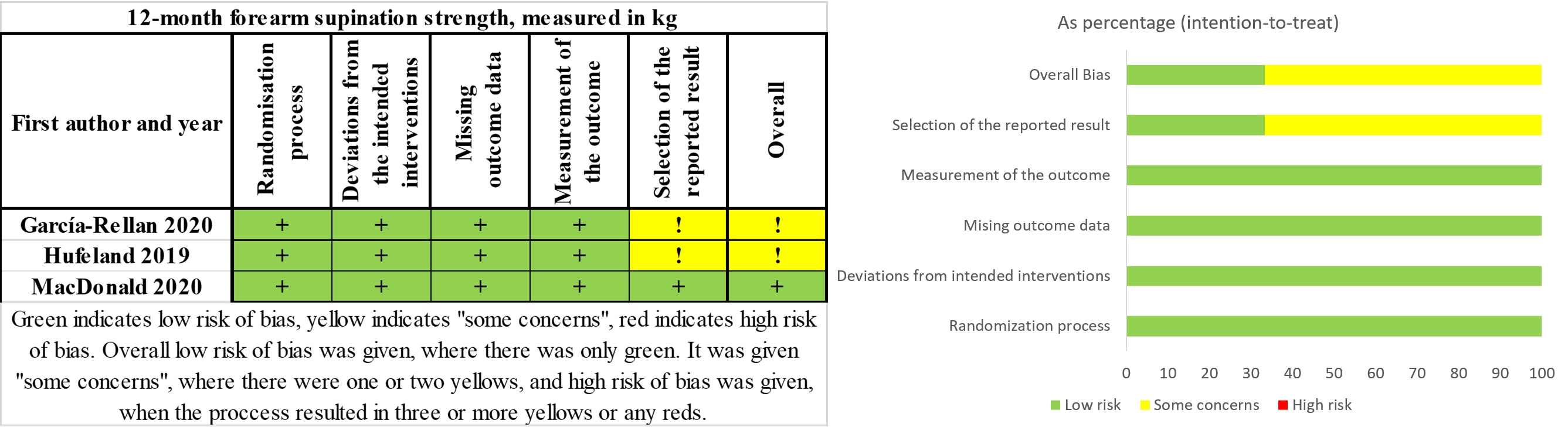

Supplement: Supplementary file 22 — Supplementary Fig. 21 Risk of bias assessment of the 12-month forearm supination strength outcome, measured in kilogram (kg) (PNG 176 KB) [file 264_2022_5338_MOESM22_ESM.png]

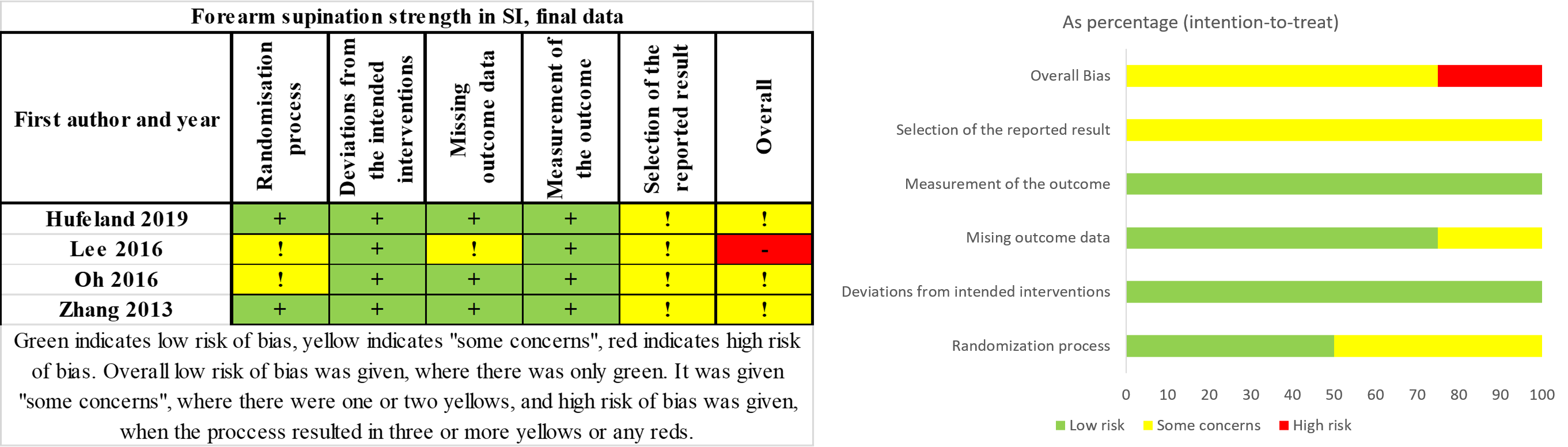

Supplement: Supplementary file 23 — Supplementary Fig. 22 Risk of bias assessment of the final forearm supination strength outcome, measured in Strength Index (SI) (PNG 179 KB) [file 264_2022_5338_MOESM23_ESM.png]

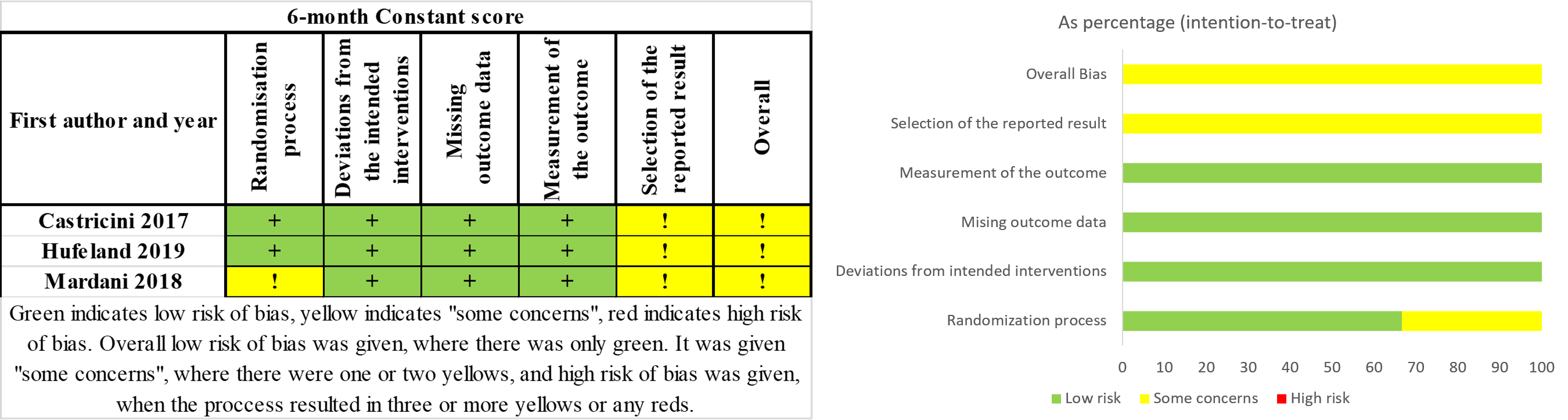

Supplement: Supplementary file 24 — Supplementary Fig. 23 Risk of bias assessment of the 6-month Constant score outcome (PNG 169 KB) [file 264_2022_5338_MOESM24_ESM.png]

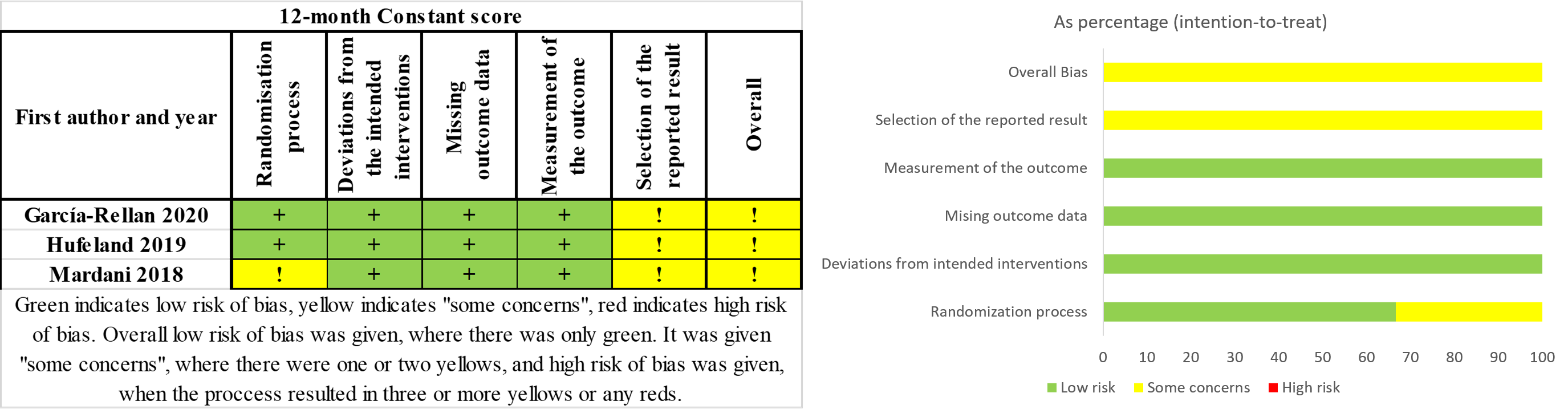

Supplement: Supplementary file 25 — Supplementary Fig. 24 Risk of bias assessment of the 12-month Constant score outcome (PNG 170 KB) [file 264_2022_5338_MOESM25_ESM.png]

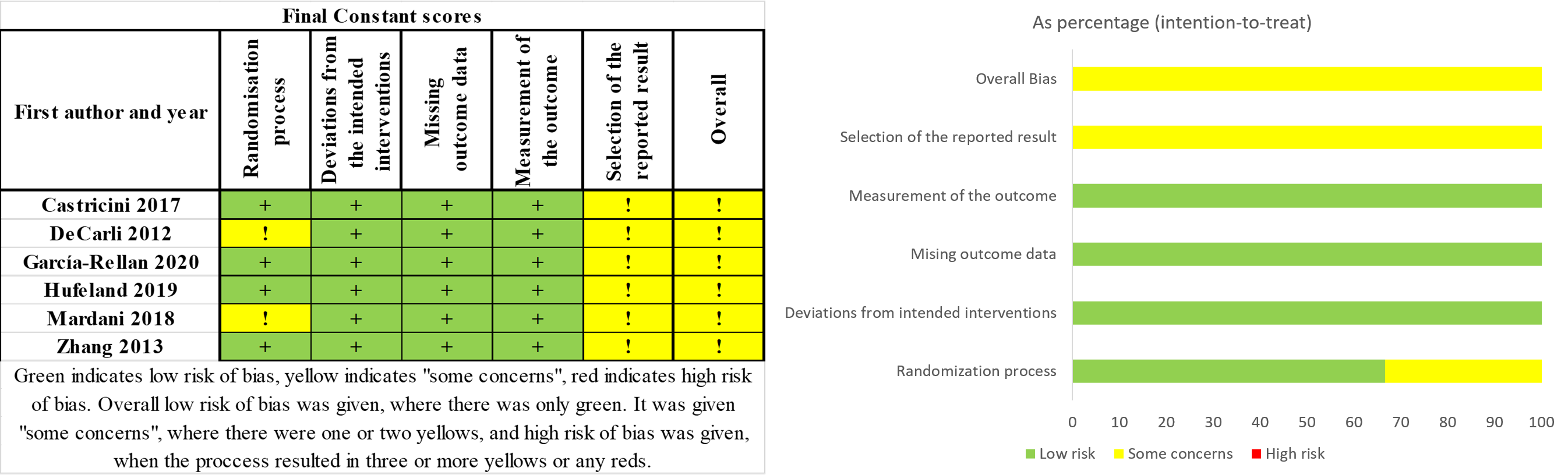

Supplement: Supplementary file 26 — Supplementary Fig. 25 Risk of bias assessment of the final Constant score outcome (PNG 179 KB) [file 264_2022_5338_MOESM26_ESM.png]

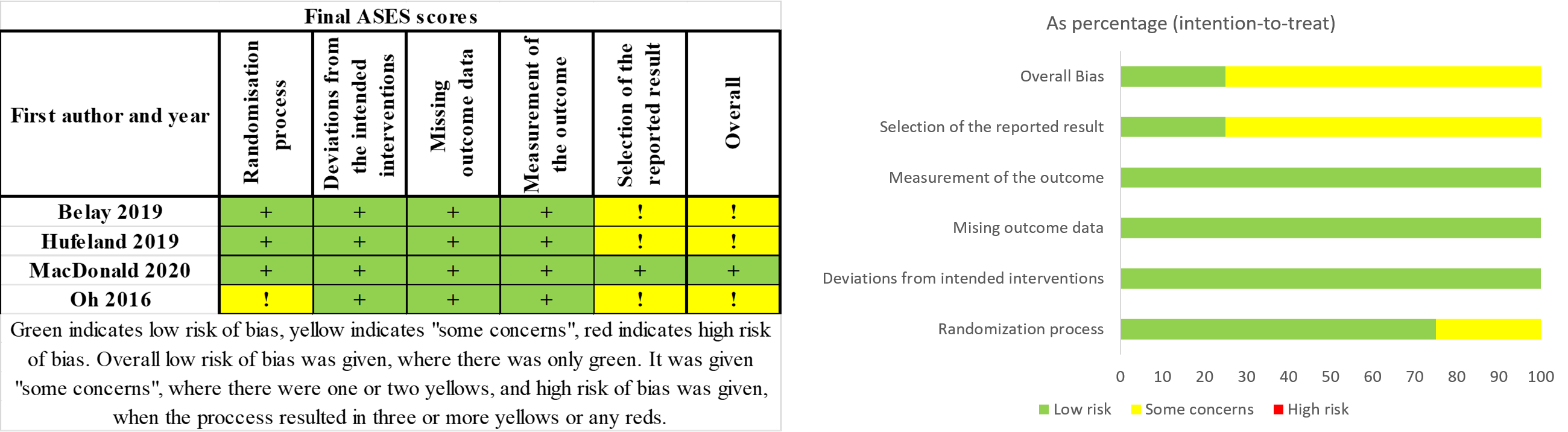

Supplement: Supplementary file 27 — Supplementary Fig. 26 Risk of bias assessment of the final American Shoulder and Elbow Surgeons (ASES) score outcome (PNG 160 KB) [file 264_2022_5338_MOESM27_ESM.png]

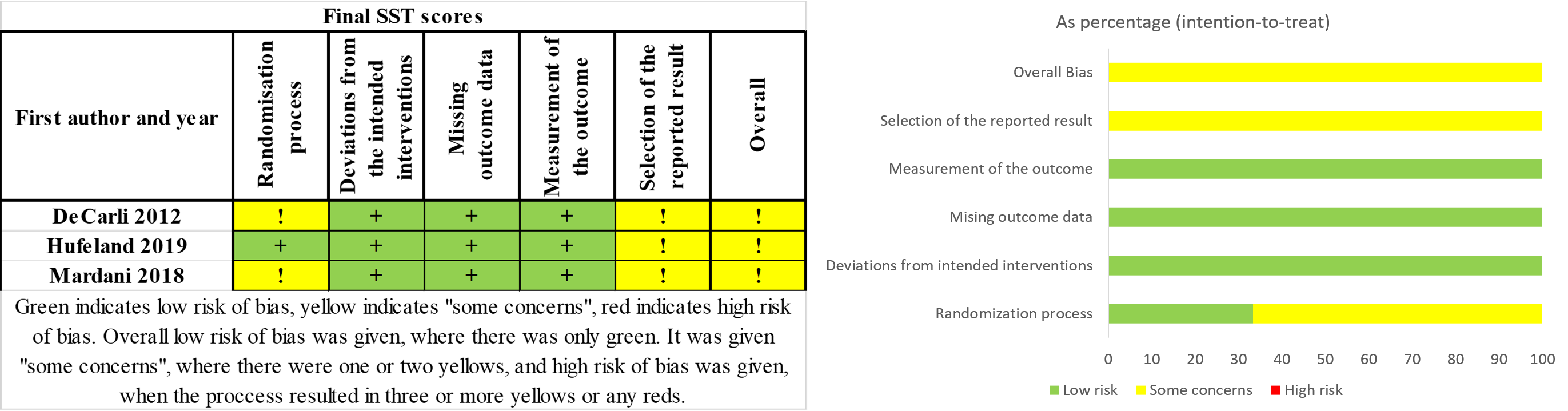

Supplement: Supplementary file 28 — Supplementary Fig. 27 Risk of bias assessment of the final Simple Shoulder Test (SST) score outcome (PNG 168 KB) [file 264_2022_5338_MOESM28_ESM.png]

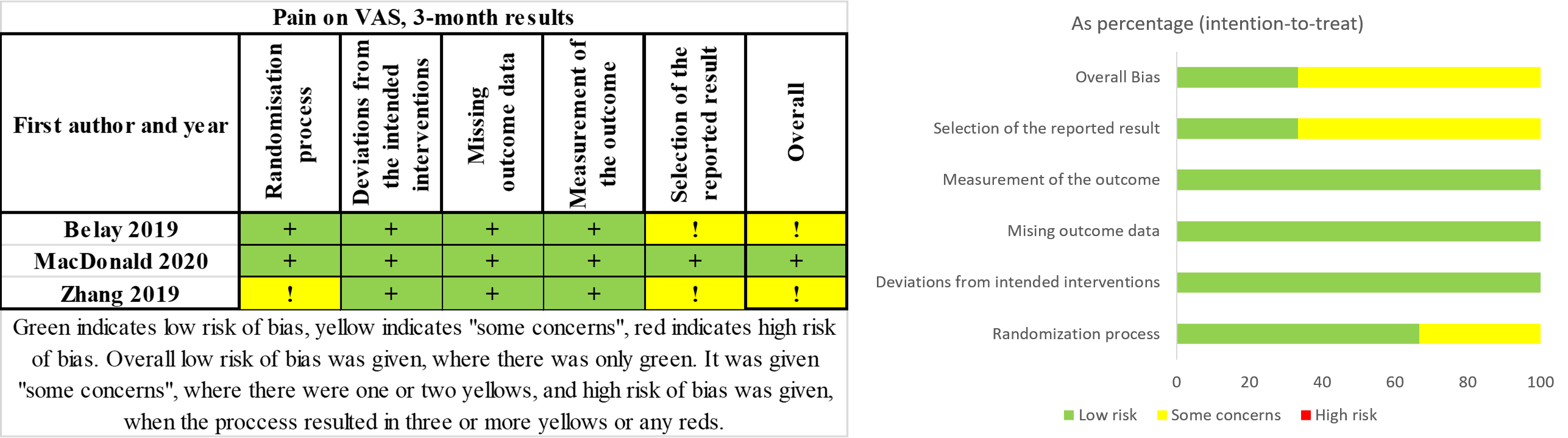

Supplement: Supplementary file 29 — Supplementary Fig. 28 Risk of bias assessment of the 3-month pain levels on the Visual Analog scale (VAS) outcome (PNG 168 KB) [file 264_2022_5338_MOESM29_ESM.png]

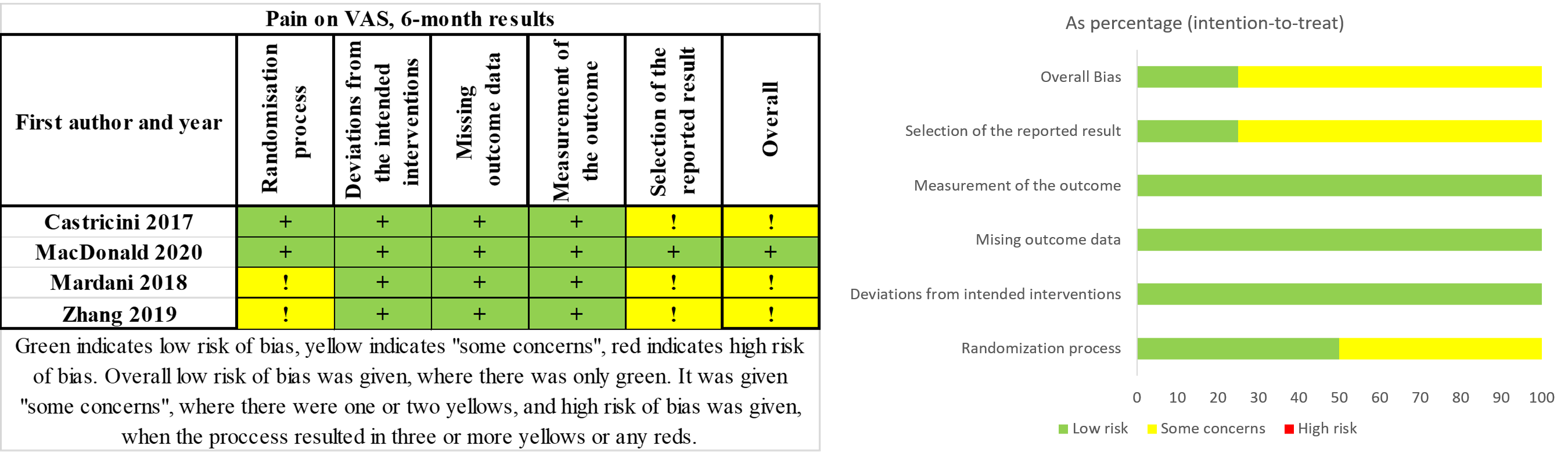

Supplement: Supplementary file 30 — Supplementary Fig. 29 Risk of bias assessment of the 6-month pain levels on the Visual Analog scale (VAS) outcome (PNG 180 KB) [file 264_2022_5338_MOESM30_ESM.png]

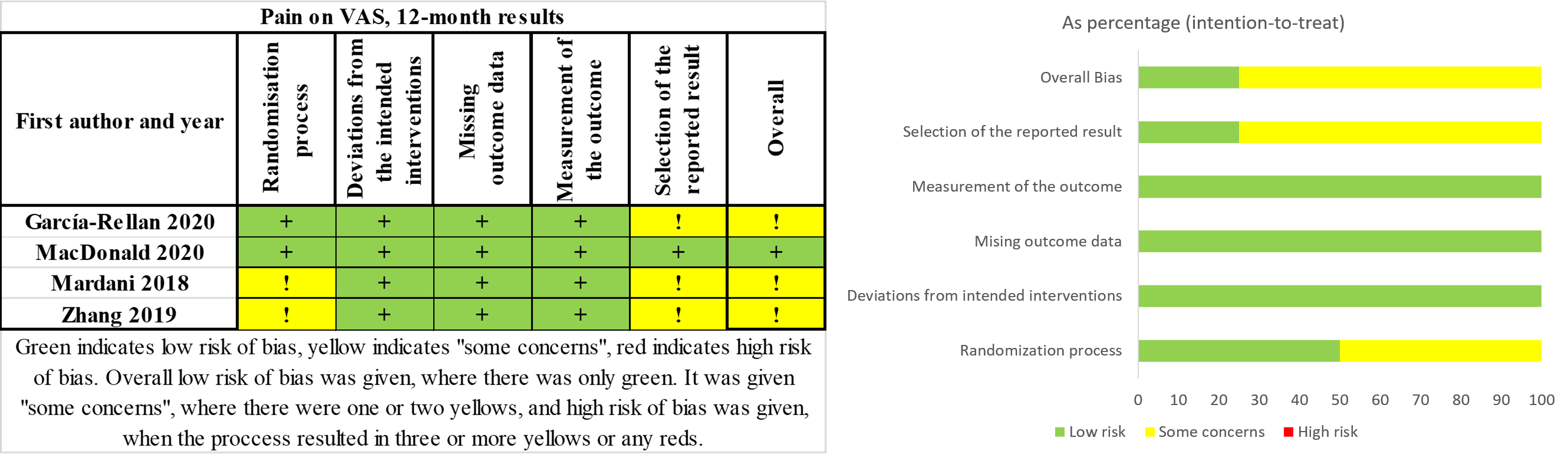

Supplement: Supplementary file 31 — Supplementary Fig. 30 Risk of bias assessment of the 12-month pain levels on the Visual Analog scale (VAS) outcome (PNG 179 KB) [file 264_2022_5338_MOESM31_ESM.png]

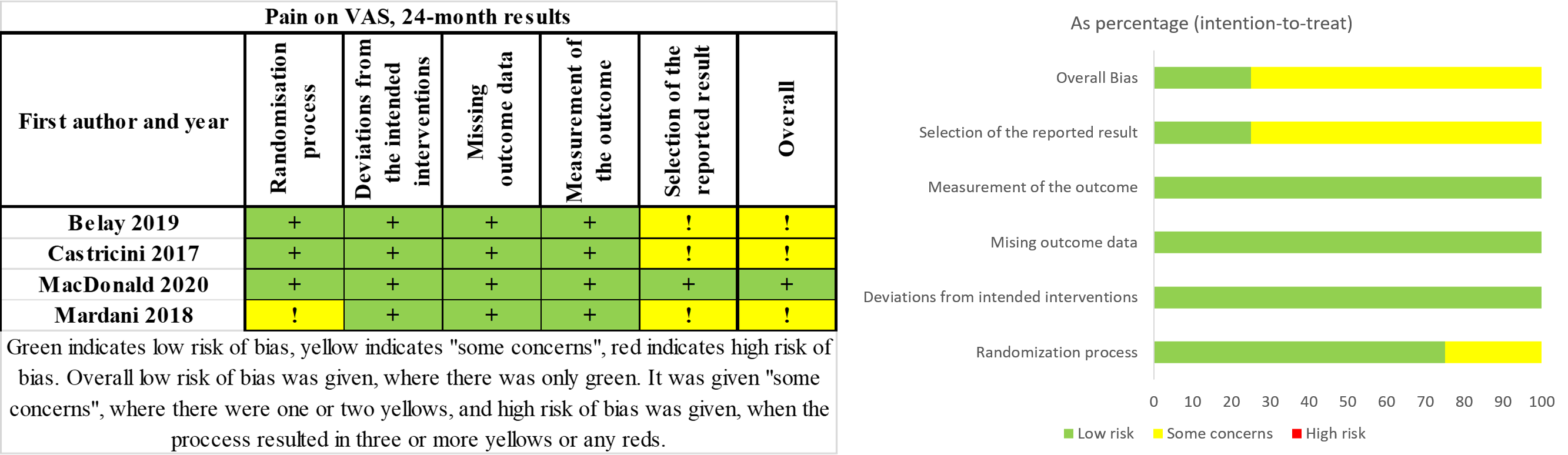

Supplement: Supplementary file 32 — Supplementary Fig. 31 Risk of bias assessment of the 24-month pain levels on the Visual Analog scale (VAS) outcome (PNG 180 KB) [file 264_2022_5338_MOESM32_ESM.png]

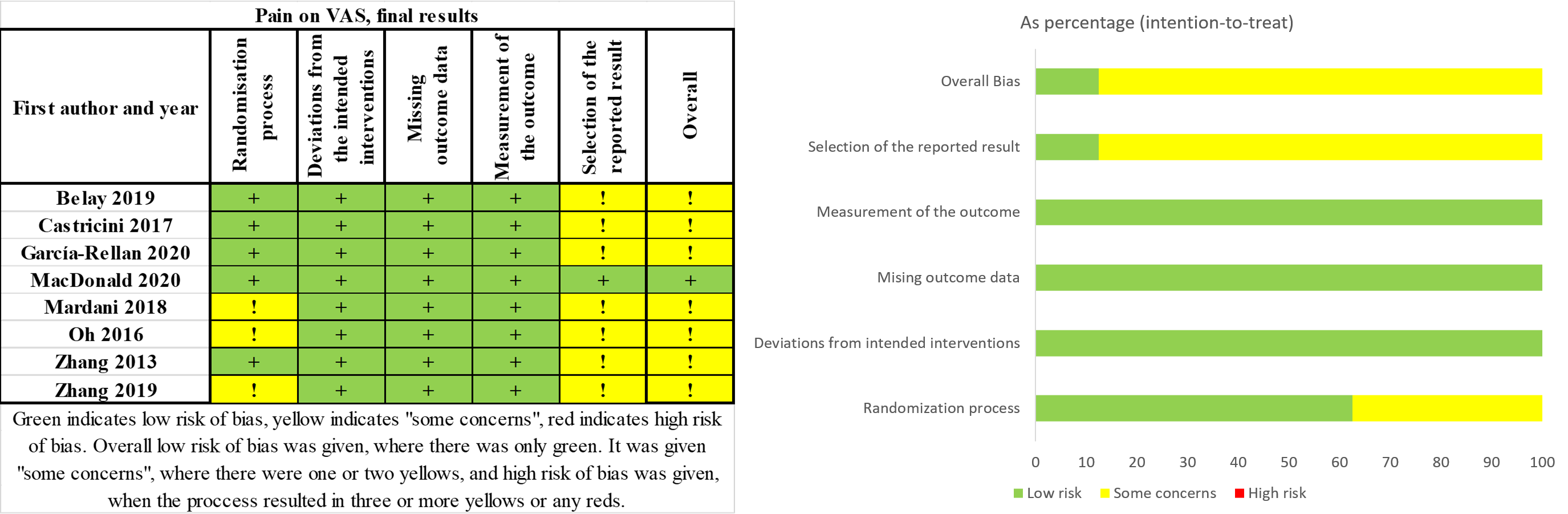

Supplement: Supplementary file 33 — Supplementary Fig. 32 Risk of bias assessment of the final pain levels on the Visual Analog scale (VAS) outcome (PNG 193 KB) [file 264_2022_5338_MOESM33_ESM.png]

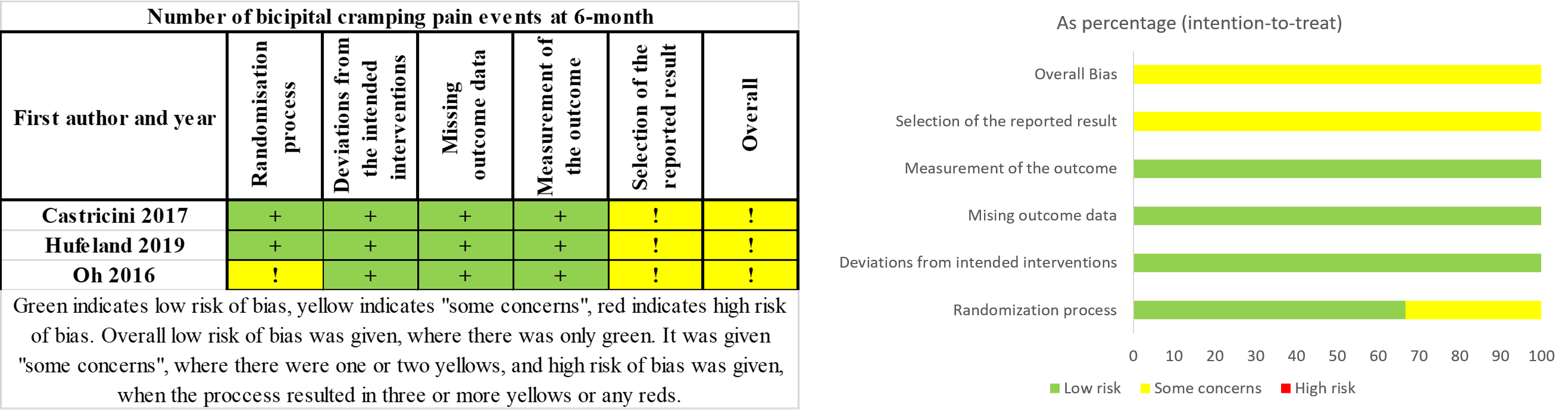

Supplement: Supplementary file 34 — Supplementary Fig. 33 Risk of bias assessment of the number of bicipital cramping pain events at 6 months outcome (PNG 163 KB) [file 264_2022_5338_MOESM34_ESM.png]

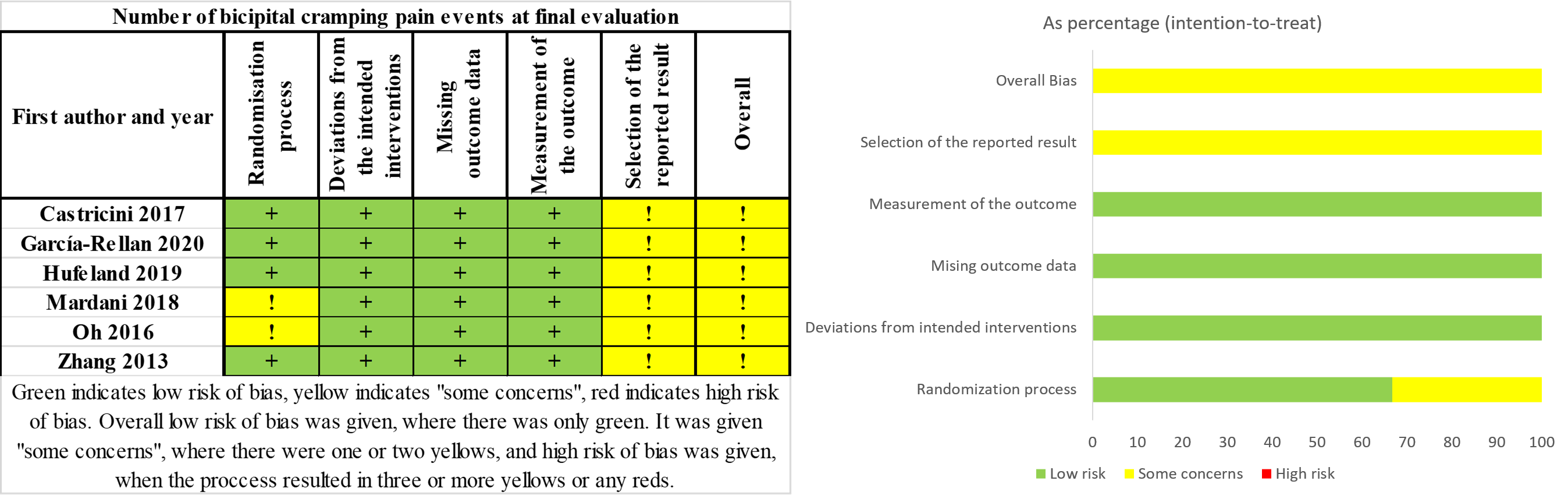

Supplement: Supplementary file 35 — Supplementary Fig. 34 Risk of bias assessment of the number of bicipital cramping pain events at the final evaluation outcome (PNG 195 KB) [file 264_2022_5338_MOESM35_ESM.png]

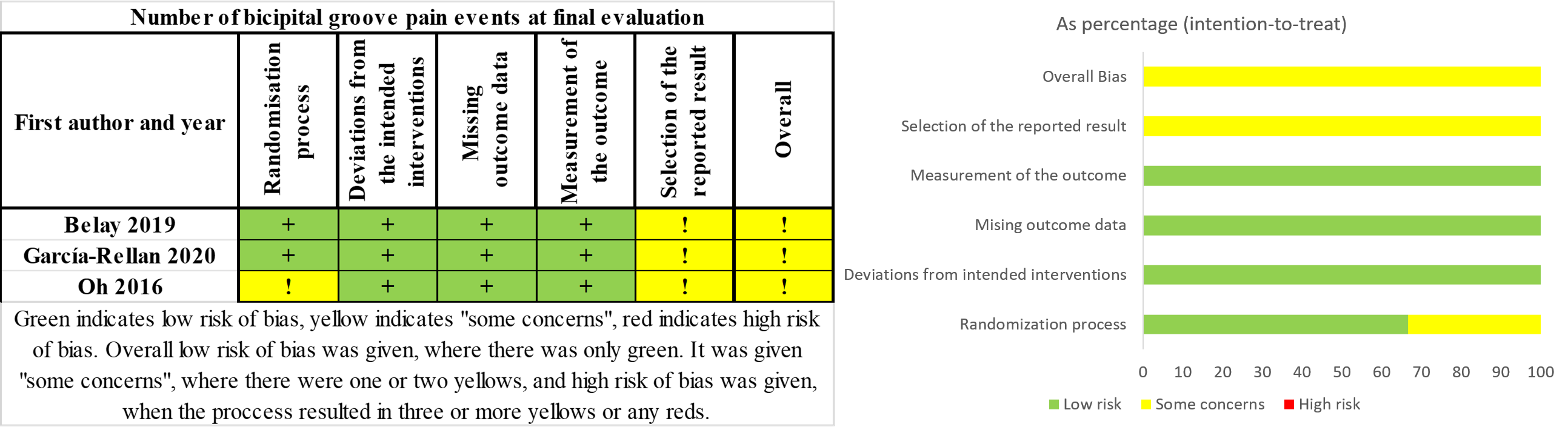

Supplement: Supplementary file 36 — Supplementary Fig. 35 Risk of bias assessment of the number of bicipital groove pain events at the final evaluation outcome (PNG 168 KB) [file 264_2022_5338_MOESM36_ESM.png]

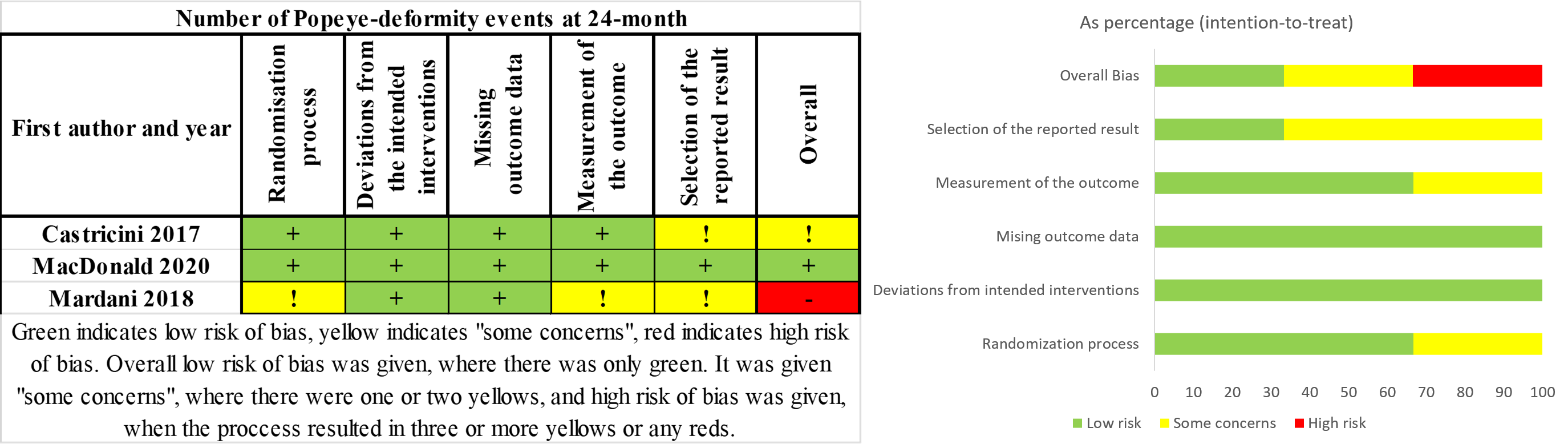

Supplement: Supplementary file 37 — Supplementary Fig. 36 Risk of bias assessment of the number of Popeye deformity events at 24 months outcome (PNG 190 KB) [file 264_2022_5338_MOESM37_ESM.png]

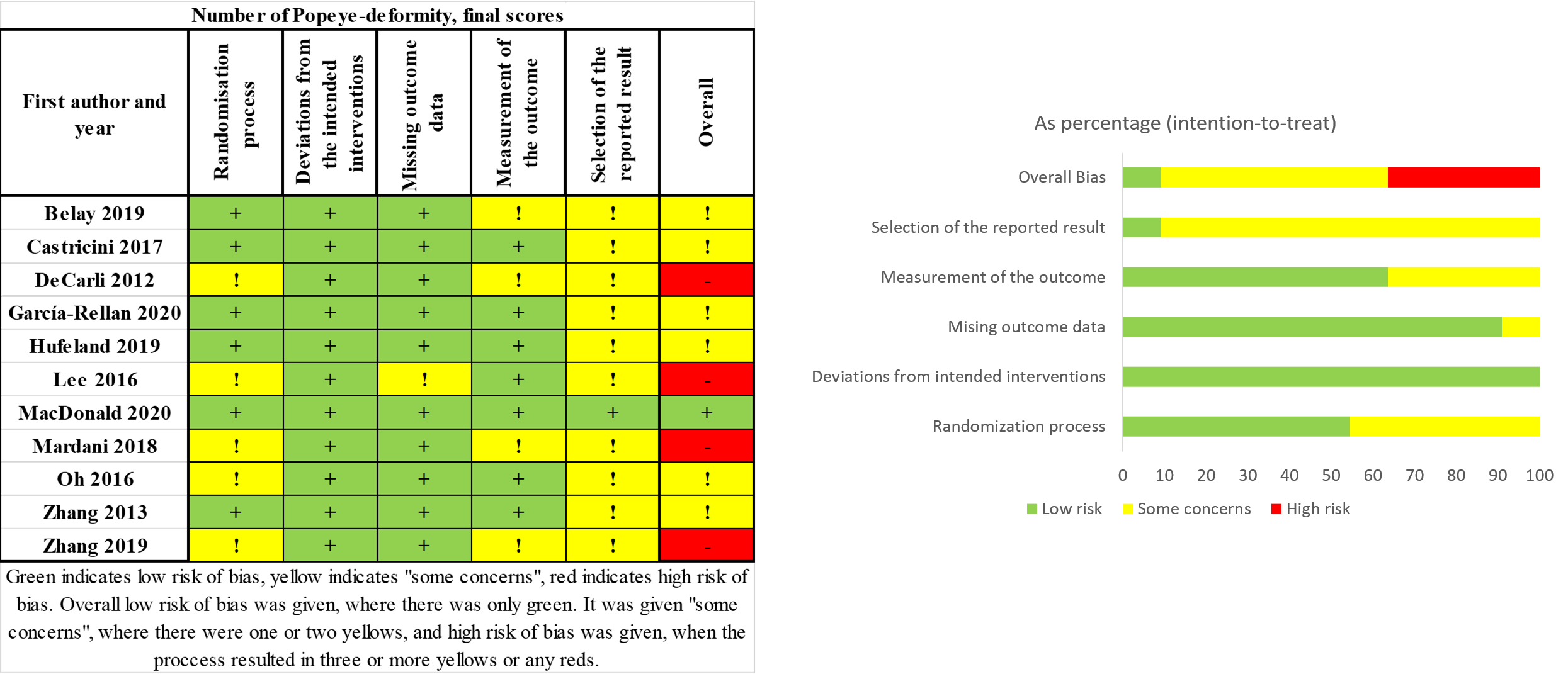

Supplement: Supplementary file 38 — Supplementary Fig. 37 Risk of bias assessment of the number of Popeye deformity events at the final evaluation outcome (PNG 207 KB) [file 264_2022_5338_MOESM38_ESM.png]

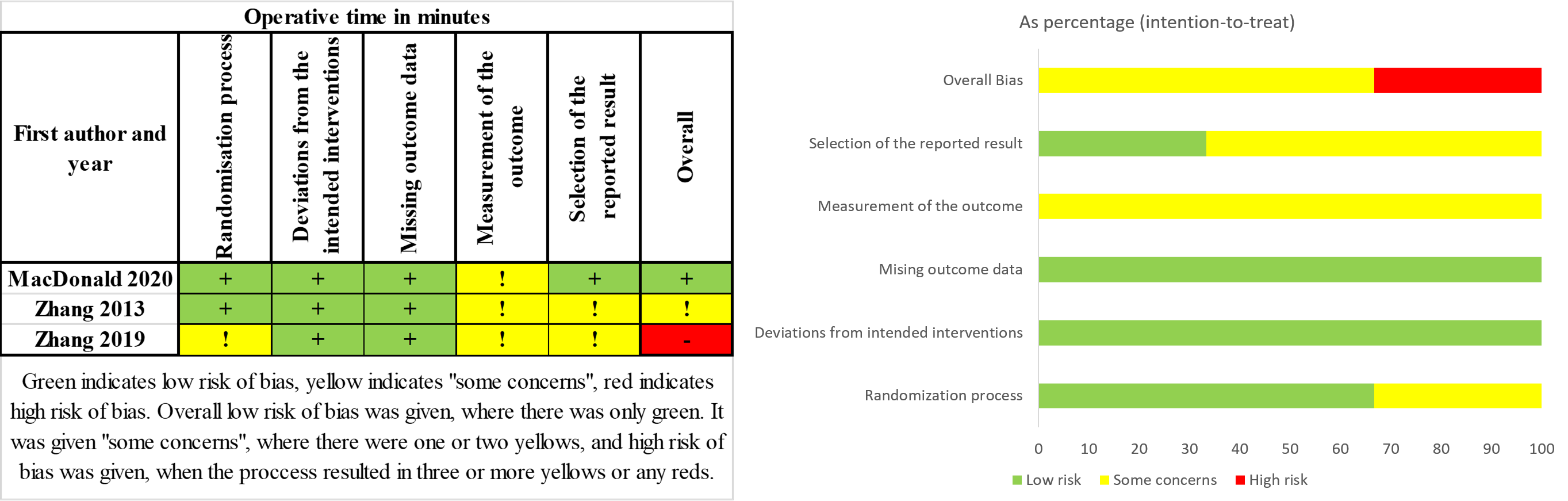

Supplement: Supplementary file 39 — Supplementary Fig. 38 Risk of bias assessment of the operative time outcome measured inminutes (PNG 180 KB) [file 264_2022_5338_MOESM39_ESM.png]
